# Compartmental Surgery With Microvascular Free Flap Reconstruction in Patients With T1–T4 Squamous Cell Carcinoma of the Tongue: Analysis of Risk Factors, and Prognostic Value of the 8th Edition AJCC TNM Staging System

**DOI:** 10.3389/fonc.2020.00984

**Published:** 2020-07-14

**Authors:** Filippo Carta, Daniela Quartu, Cinzia Mariani, Melania Tatti, Valeria Marrosu, Edoardo Gioia, Clara Gerosa, Jacopo S. A. Zanda, Natalia Chuchueva, Andrea Figus, Roberto Puxeddu

**Affiliations:** ^1^Unit of Otorhinolaryngology, Department of Surgery, Azienda Ospedaliero-Universitaria di Cagliari, University of Cagliari, Cagliari, Italy; ^2^Unit of Pathology, Department of Surgery, Azienda Ospedaliero-Universitaria di Cagliari, University of Cagliari, Cagliari, Italy; ^3^ENT Department, I. M. Sechenov First Moscow State Medical University, Moscow, Russia; ^4^Unit of Plastic Surgery, Department of Surgery, Azienda Ospedaliero-Universitaria di Cagliari, University of Cagliari, Cagliari, Italy

**Keywords:** tongue cancer, compartmental surgery, head and neck, free flaps, American Joint Committee on Cancer

## Abstract

Compartmental surgery and primary reconstruction with microvascular free flaps represent the gold-standard in the treatment of oral tongue squamous cell carcinoma (OTSCC). However, there are still unclear clinical features that negatively affect the outcomes. This retrospective study included 80 consecutive patients with OTSCC who underwent compartmental surgery and primary reconstruction by free flap. The oncologic outcomes, the reliability of the 8th edition American Joint Committee on Cancer (AJCC) staging system and the prognostic factors were evaluated. Fifty-nine males and 21 females (mean age 57.8 years, range 27–81 years) were treated between November 2010 and March 2018 (one patient had two metachronous primaries). Seventy-one patients (88.75%, 52 males, 19 females, mean age of 57.9 years, range of 27–81 years) had no clinical history of previous head and neck radiotherapy and were considered as naive. Histology showed radical surgery on 80/81 lesions (98.8%), with excision margins >0.5 cm, while in 1 case (1.2%), a close posterior margin was found. According to the 8th AJCC classification, 37 patients (45.7%) were upstaged shifting from the clinical to the pathological stage, and 39 (48.1%) showed an upstaging while shifting from the 7th to the 8th AJCC staging system (no tumors were downstaged). Nodal involvement was confirmed in 33 patients (40.7%). Perineural and lymphovascular invasion were present in 9 (11.1%) and 11 (13.6%) cases, respectively. Twenty-two patients (27.1%) underwent adjuvant therapy. The 5-years disease-specific, overall, overall relapse-free, locoregional relapse-free and distant metastasis-free survival rates were 73.2, 66.8, 62.6, 67.4, and 86%, respectively. Patients with a lymph node ratio >0.09 experienced significantly worse outcomes. Univariate analysis showed that patients with previous radiotherapy, stage IV disease, nodal involvement, and lymphovascular invasion had significantly worse outcomes. Multivariate analysis focused naive patients and showed that lymphovascular invasion, advanced stage of disease, and node involvement resulted reliable prognostic factors, and patients with the same tumor stage and histological risk factors who did not undergo adjuvant therapy experienced significantly worse outcomes. In our series, surgery played a major role in the treatment of local extension; adjuvant therapy resulted strictly indicated in patients with advanced-stage disease associated with risk factors.

## Introduction

The incidence of oral tongue squamous cell carcinoma (OTSCC) is currently estimated at 5.21/100,000 population, and ~3.06/100,000 new cases per year are documented in Italy (1–3).

OTSCC is classically associated with the main risk factors for all squamous cell carcinomas of the upper aerodigestive tract, such as smoking, alcohol consumption, human papillomavirus (HPV), environmental factors (chemical and physical), diet, and occupation (4).

The prognosis of OTSCC in patients with advanced disease is generally poor; in addition, patients with T1-T2N0 disease experience a greater than expected rate of regional and locoregional relapse if inadequately treated (5).

In recent decades, oncologic outcomes of patients with OTSCC have improved due to the introduction of two main concepts: anatomy-based compartmental tongue surgery (CTS) and the systematic reconstruction of oral defects by microvascular free flaps. The principles of compartmental surgery advocate the removal of compartments (anatomic-functional units) containing the primary tumor, with the excision of the lesion along with the potential muscular, vascular, nervous, and lymphatic pathways that may lead to spread and recurrence (6). The diffusion of CTS has been facilitated by the increasing popularity of microvascular free flap reconstruction because three-dimensional radical resection cannot be performed without the reconstruction that allows the restoration of important functions of the tongue, such as voice articulation, swallowing and breathing. The improvement of disease local control after CTS has been significant (6), positively affecting prognosis and locoregional spread and allowing a better understanding of important prognostic factors (7). As a result, the revision of the 7th edition American Joint Committee on Cancer (AJCC) TNM staging system included depth of invasion (DOI) and extranodal extension (ENE) as fundamental predictors of disease-specific survival (DSS), providing a more reliable prognosis (8, 9).

However, despite the improvement in the understanding and management of OTSCC, there are still unclear clinical features that negatively impact the locoregional control and the incidence of distant metastasis that should be better understood.

Therefore, the authors performed a retrospective/prospective study of 80 patients consecutively treated for OTSCC with the aim of evaluating the oncologic outcomes after CTS and contemporary reconstruction with microvascular free flap. A comparison of the prognostic reliability of the two TNM staging systems (AJCC 2010 vs. AJCC 2017) and an evaluation of clinical and histological features were performed. An additional objective was to evaluate the weight of adjuvant therapy based on particular histological and clinical findings.

## Materials and Methods

The present study was approved by the Ethics Committee “Commissione del Comitato Etico Indipendente della Azienda Ospedaliero-Universitaria di Cagliari” (NP/2018/895).

All consecutive patients who underwent CTS and microvascular reconstructive surgery with curative intent between November 2010 and March 2018 for OTSCC at any stage of disease were included. Patients with previous chemotherapy (CHT) and/or radiotherapy (RT) were also included in the enrollment but the analysis focused patients without previous RT for head and neck malignancies who were considered “naive.” Patients with a clinical history of previous transoral surgery alone performed elsewhere were considered as naive since, in such cases, the CTS allows a resection including the relapsed lesion and the surrounding scar tissue.

Eligible for CTS were patients affected by OTSCC more than 2 cm in greatest dimension or with more than 5 mm of DOI at computer tomography (CT) and/or magnetic resonance imaging (MRI) and extended also to pelvis and mandible. CTS was also performed in cT1 tumors when the epicentrum of the lesion was localized in the posterior and lateral aspect of the tongue or in case of any proximity of the tumor to the paramedian and/or lateral septum. Patients with contraindications for microvascular procedures, such as advanced arteriosclerosis underwent pedicled flap reconstruction and were not included in the present study.

Correlations of age and comorbidities were established according to the Age Adjusted Charlson Comorbidity Index (AACCI) (10–14).

**Figure 1:**
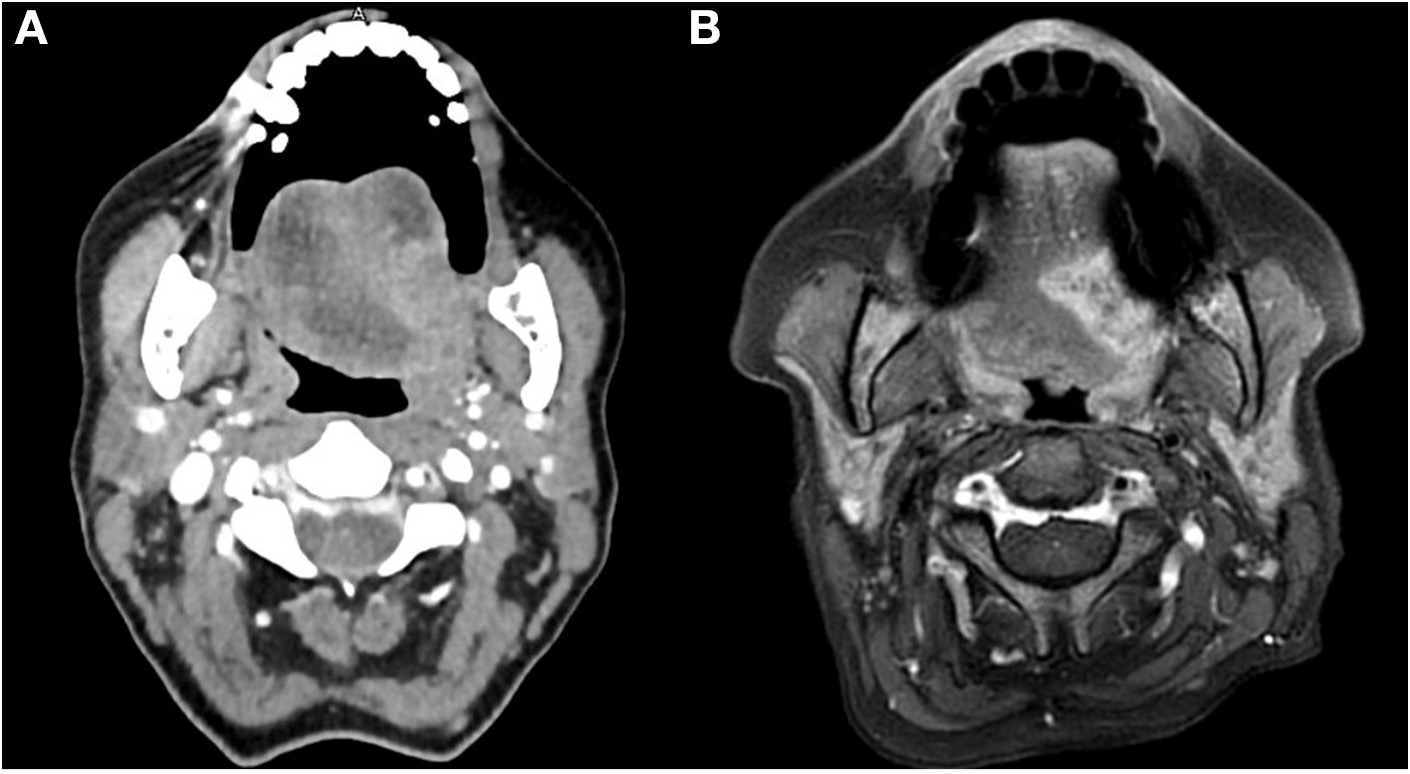
CT **(A)** and MRI **(B)** preoperative evaluation of a patient with OTSCC. The image shows the higher definition of the boundaries of the tumor obtained with the MRI.

Preoperative head and neck CT and MRI (from 2013 the MRI method has been routinely preferred since considered more accurate during the preoperative evaluation as shown in Figure 1), total body PET-CT (in case of relapse/persistence of disease), and color Doppler ultrasound of neck vessels and free-flap donor vessels (to evaluate anatomy and caliber of the vessels with the perforator's anatomy) were routinely performed.

From 2013 the superficial spread of the tumor was assessed with the Narrow Band Imaging (Olympus Medical System Corporation Tokyo, Japan), and subsequently with the IMAGE 1S (Karl Storz, Tuttlingen, German), with 0 and 30° rigid endoscope (Andrea-Dias Contact Micro Laryngoscope, Karl Storz, Tuttlingen, Germany and Hamou Micro Contact Hysteroscope, Karl Storz, Tuttlingen, Germany) (15).

Preoperative histologic diagnosis was obtained for all patients.

All patients were clinically and pathologically staged according to the 7th and subsequently to the 8th edition of the AJCC TNM staging system and classified with clinical (preoperative) TNM and pathological (post-operative) TNM (8, 9, 16, 17).

Authors performed a precise retrospective analysis from a histopathological perspective, nevertheless the CTS was applied to all patients with tongue cancer in a prospective way from 2010. The analysis on the basis of the 2017 AJCC classification was performed by a dedicate pathologist to all surgical specimens of all patients treated since 2010.

All patients underwent compartmental radical excision of the primary tumor transorally, median mandibulotomy or with a pull-through approach, with ipsilateral or bilateral, selective or radical/modified radical neck dissection (according to the site of the tumor to the midline and the clinical neck node status).

Surgical procedures were classified according to Ansarin et al. (18): type I glossectomy (mucosectomy), type II glossectomy (partial glossectomy), type IIIa glossectomy (hemiglossectomy), type IIIb glossectomy (compartmental hemiglossectomy), type IVa glossectomy (subtotal glossectomy), type IVb glossectomy (near-total glossectomy), and type V glossectomy (total glossectomy).

Surgical resections were performed following an anatomic-based strategy: unilateral resections were extended from the lingual septum (the medial margin) to the pelvis or mucosa of the mandible (the lateral margin), the stylohyoid muscle (posterior margin), and the mylohyoid muscle, which was considered the floor of the compartment (type III glossectomy). Lesions involving both sides of the tongue were resected from pelvis to pelvis and inferiorly to the hyoid bone (type IV–V glossectomy). Patients with mandibular involvement underwent a wider resection, including the corresponding mandibular bone (marginal or segmental mandibulectomy).

All surgical defects of the oral cavity were primarily reconstructed with microvascular free flaps. The radial forearm (RF) and anterolateral thigh (ALT) free flaps were the first choice for intraoral reconstruction. Partial glossectomy was reconstructed with RF or perforator ALT flaps, total glossectomy was reconstructed with an ALT free flap or vertical rectus abdominis myocutaneous (VRAM) free flap, and the composite bony iliac crest deep circumflex iliac artery (DCIA) free flap was the procedure of choice in cases in which glossectomy was associated with segmental mandibulectomy to reconstruct composite intra-oral defects since the cutaneous and muscular component of this flap (external oblique, internal oblique and transverse muscles) provides abundant tissue for the reconstruction.

Anastomoses were performed using an operative microscope (ZEISS S7 Microscope, Carl Zeiss, USA; focal length 250 mm). Arterial anastomosis was performed with synthetic non-absorbable 8/0 or 9/0 nylon sutures. Venous anastomosis was performed with a coupler device (Microvascular Anastomotic Coupling System, Synovis Life Technologies). All patients received a single bolus of heparin sodium (1,500 IU) at least 5 min before the transfer of the flap.

Temporary tracheostomy was performed in all patients to avoid post-operative respiratory distress.

Intraoperative and post-operative fluid balance was routinely evaluated with the goal of maintaining intravascular fluid volume for optimal tissue blood flow and oxygenation (19).

All patients had nasogastric feeding tube inserted, which was kept in place until acceptable swallowing function was restored. After 30 post-operative days, percutaneous endoscopic gastrostomy (PEG) was indicated in cases with inadequate post-operative swallowing function.

Post-operative treatment consisted of the antibiotic protocol for the head and neck (ceftriaxone 2 g/day iv and metronidazole 500 mg 3 times/day iv for 7–10 days), and low molecular weight heparin (enoxaparin sodium, range of 3,000–8,000 IU/day) associated with an antiembolism stocking for the prophylaxis of deep venous (DVT) and microvascular. An Ear Nose and Throat specialist-in-training monitored the free flap every hour during the first 48 h and every 4 h up to 5 post-operative days according to the internal protocol to detect early signs of vascular impairment that could require surgical exploration/revision of the anastomosis.

Hospitalization time and complications were evaluated. According to the Clavien-Dindo System (20) and Genden et al. (21). Complications were divided into surgical donor-site and flap complications, which require surgical revision, and non-surgical donor-site and flap complications, which were treated with medical therapy. Donor-site complications consisted of hematoma, seroma, infection, wound dehiscence, venous congestion and skin loss; flap complications included partial or total flap failure, cervical hematoma, infection, wound dehiscence, and fistula. Additionally, systemic complications were documented and included post-operative hypertension (PH), post-operative arrhythmia (PA), myocardial infarction (MI), pulmonary edema (PO), pulmonary embolism (PE), DVT, acute renal failure (ARF), respiratory distress (RD), pneumonia and sepsis.

Perineural invasion (PNI), lymphovascular invasion (LVI), DOI and ENE were evaluated in all patients. The ratio between the positive and overall number of removed nodes was calculated as the lymph node ratio (LNR), which was considered a prognostic value when higher than 0.09 (22).

Adjuvant RT was planned in cases with pT3–pT4 lesions, close margins, multiple nodal involvement, and neural, lymphatic and/or vascular invasion (23).

All patients were included in our post-operative follow-up, planned according to the American Head & Neck Society (AHNS) guidelines (24). Disease-free was defined as the absence of persistence or recurrence as demonstrated by a clinical examination performed by an experienced Head and Neck surgeon, with imaging followed by histopathology if needed. The definition of evidence of disease referred to the presence of a local, regional or locoregional relapse that was histologically proved and/or distant metastases.

Recurrence time was assessed from the date of surgery to the date of the first recurrence. The 5-years DSS, overall survival (OS), overall relapse-free survival (ORFS), local relapse-free survival (LRFS), and distant metastasis-free survival (DMFS) were calculated using the Kaplan-Meier method. Statistical analysis was performed using GraphPad Prism software (GraphPad, San Diego, CA, USA). Univariate analysis was performed to determine the statistical significance of the oncologic results observed according to different risk factors/clinical features (stage, previous treatments, neural, lymphatic and/or vascular invasion, and adjuvant treatments) in all patients and in naive patients (those without a clinical history of previous head and neck RT); multivariate analysis focused only naive patients to remove the possible bias due to the changes in lymphatic and vascular network induced by RT that could make outcomes unpredictable; statistical significance was defined as *p* < 0.05.

According to Cramer et al. (25) we evaluated five quality metrics for our series: negative surgical margins, neck dissection yielding 18 nodes, appropriateness of adjuvant RT indication, appropriateness of adjuvant CHT-RT indication, and timing of adjuvant CHT-RT.

## Results

Fifty-nine males and 21 females (mean age of 57.8 years, range of 27–81 years) were definitively enrolled in the present study. Seventy-one patients (88.75%, 52 males, 19 females, mean age of 57.9 years, range of 27–81 years) had no clinical history of previous hand and neck radiotherapy (naive patients), and, among them, one patient (1.25%) underwent CTS with primary free flap reconstruction for two metachronous primaries, and 2 patients were treated for recurrent OTSCC treated elsewhere with conventional transoral surgery alone. Three patients (3.7%) were treated after failure of CHT-RT, and six procedures (7.4%) were performed in patients with recurrent disease who were previously treated elsewhere with surgery and adjuvant RT.

According to the 8th AJCC staging system, 19 patients (23.5%) were staged as cT1, 48 patients (59.3%) as cT2, 1 patient (1.2%) as cT3, and 13 patients (16%) as cT4a. Fifty-two patients (64.2%) were staged as cN0, 11 patients (13.6%) as cN1 and 18 patients (22.2%) as cN2. Eighteen patients (22.2%) had malignancies classified as stage I, 28 patients (34.6%) as stage II, 9 patients (11.1%) as stage III, 25 patients (30.9%) as stage IVA, and 1 patient (1.2%) as stage IVC.

Patients underwent 81 surgical procedures: 65 type IIIb glossectomy (80.3%), 7 type IVa glossectomy (8.6%), 5 type IVb glossectomy (6.2%), and 4 type V glossectomy (4.9%). Resection of the mandible was necessary in 7 patients (8.6%): marginal mandibulotomy in 4 cases (4.9%) and segmental mandibulectomy in 3 cases (3.7%). A total of 19 patients (23.5%) underwent CTS through a transmandibular approach with a lower lip splitting incision, and 55 patients (67.9%) were treated by a combined transoral and transcervical approach without mandibular splitting.

A total of 57 patients (70.4%) underwent unilateral neck dissection, and 22 (27.1%) underwent bilateral neck dissection. Fifty-seven patients (56.4%) underwent selective neck dissections (SNDs), of which the removal of I-III levels was performed in 19 patients (18.8%), the removal of I-IV levels was performed in 35 patients (34.6%), and the removal of II–V levels was performed in 3 patients (3%). A total of 41 patients (40.6%) underwent type III modified radical neck dissections (MRNDs), 2 patients (2%) underwent type I MRND, and 1 patient (1%) underwent type II MRND. Two patients (2.5%) with a clinical history of previous neck dissection underwent CTS solely followed by microvascular reconstruction with neck dissection limited to the area of the vascular pedicle. The age distribution, procedures, reconstruction and histology are detailed in Table 1.

**Table 1:** Patients' age distribution, site of reconstruction and histology.

**Age**		**No. of cases/Frequency%**
All patients	Mean age 57.8 years	80
	Range 27–81 years	
	Younger: <65 years	58/72.5
	Young old: 65–74 years	16/20
	Older and oldest old: ≥75 years	6/7.5
Male	Mean age 56.7 years	59/73.8
	Range 27–79 years	
Female	Mean age 61.3 years	21/26.2
	Range 42–81 years	
Naive patients	Mean age 57.9 years	71
	Range 27–81 years	
	Younger: <65 years	52/73.3
	Young old: 65–74 years	14/19.7
	Older and oldest old: ≥75 years	5/7
Male	Mean age 57.1 years	52/73.2
	Range 27–79 years	
Female	Mean age 61.1 years	19/26.8
	Range 42–81 years	
	**Oral cavity procedures**	**No. of procedures/Frequency%**
All patients	Type IIIb glossectomy	65/80.3
	+ Marginal mandibulotomy	4/4.9
	+ Segmental mandibulectomy	1/1.2
	Type IVa glossectomy	7/8.6
	+ Segmental mandibulectomy	3/2.5
	Type IVb glossectomy	5/6.2
	Type V glossectomy	4/4.9
	All procedures	81^*^
Naive	Type IIIb glossectomy	61/84.7
	+ Marginal mandibulotomy	4/5.6
	+ Segmental mandibulectomy	1/1.4
	Type IVa glossectomy	6/8.3
	+ Segmental mandibulectomy	3/4.2
	Type IVb glossectomy	3/4.2
	Type V glossectomy	2/2.8
	All procedures	72^*^
	**Microvascular procedures**	**No. of cases/Frequency%**
All patients	Forearm flee flap	64/79
	ALT free flap	9/11.1
	VRAM free flap	5/6.2
	DCIA free flap	3/3.7
	All procedures	81^*^
Naïve	Forearm free flap	61/84.7
	ALT free flap	7/9.7
	VRAM free flap	3/4.2
	DCIA free flap	1/1.4
	All procedures	72^*^
	**Histology features**	**No. of cases/Frequency%**
All patient	G1	28/34.6
	G2	41/50.6
	G3	12/14.8
	p16	2/2.5
	Perineural invasion	9/11.1
	Lymphovascular invasion	11/13.6
	Perineural and lymphovascular invasion	15/18.5
	Absence of perineural/lymphovascular invasion	46/56.8
Naive	G1	26/36.1
	G2	35/48.6
	G3	11/15.3
	p16	2/2.8
	Perineural invasion	20/27.8
	Lymphovascular invasion	21/29.2
	Perineural and lymphovascular invasion	11/15.3
	Absence of perineural/lymphovascular invasion	42/58.3

* *One patient underwent two CTS procedures for two different metachronous lesions*.

All patients underwent immediate microsurgical reconstruction. RF free flap was performed in 64 cases (79%), ALT free flap in 9 cases (11.1%), VRAM free flap in 5 cases (6.2%), and DCIA free flap in 3 cases (3.7%). All free flap procedures are reported in Table 2.

**Table 2:** Microvascular free flap procedures of our series.

**Free flap procedures**	**No. of procedures/%**	**Type of glossectomy**			
		**IIIb**	**IVa**	**IVb**	**V**
Forearm	64/79	59	5	–	–
ALT	9/11.1	4	–	4	1
VRAM	5/6.2	1	–	1	3
DCIA	3/3.7	1	2	–	–
Total	81	65	7	5	4

The recipient artery for the microvascular anastomosis was the facial artery in 55 cases (67.9%), the superior thyroid artery in 24 cases (29.6%) and the lingual artery in 2 cases (2.5%). The recipient vein for the microanastomosis was one of the branches of the thyro-lingual-facial trunk in 77 cases (95.1%), followed by termino-lateral anastomosis to the internal jugular vein in 4 cases (4.9%). Venous drainage was obtained with a single anastomosis in the majority of cases (91.4%), and in all cases, it was performed with the coupler device. When a double anastomosis was performed (*n* = 7), one of the branches of the thyro-lingual-facial trunk was used in all cases, and it was coupled with the internal jugular vein in 3 cases (3.7%), with the middle thyroid vein in 3 cases (3.7%), and with the external jugular vein in 1 case (1.2%).

In 5 cases (6.2%), admission to the Intensive Care Unit (ICU) with hemodynamic and airway monitoring was considered necessary due to the chronic impairment of one or more organ systems (26).

Twenty-one patients (25.9%) experienced post-operative complications that required surgical revision in 17 cases (21%). The most experienced complication was bleeding (13 cases, 16%), followed by free flap sufferance due to venous congestion, which was managed with the revision of the anastomosis (3 cases, 3.7%), wound dehiscence (3 cases, 3.7%) and fistula (2 cases, 2.5%). No total flap failure was observed in the present series (Table 3).

**Table 3:** Complications observed in patients who underwent CTS.

**Complications**	**Forearm**	**ALT**	**VRAM**	**DCIA**	**All series%**
Flap failure	–	–	–	–	0 (0.0)
Near flap failure^*^	3	–	–	–	3 (3.7)
Cervical bleeding without flap sufferance	13	–	–	–	13 (16)
Head and neck suture dehiscence^**^	1	1	–	1	3 (3.7)
Salivary fistula^**^	1	–	1		2 (2.5)
Total	18	1	1	1	21 (25.9)

* *In 3 cases, the compression on the pedicle was associated with cervical hematoma; in 2 cases, bleeding originated from the pedicle; and in 1 case, the flap congestion was due to thrombosis of the venous pedicle*.

** *These complications were managed by a conservative approach*.

The mean time to the removal of the temporary tracheostomy was 8 days (range, 6–21 days). The mean duration of nasogastric feeding tube use was 18.1 days (range, 8–38 days), and 2 patients underwent PEG for supplemental nutrition. The mean hospitalization time was 19.3 days (range, 9–40 days).

Definitive histology showed that 80 lesions (98.8%) were completely removed with free margin, while a close posterior margin was found in 1 patient (1.2%) treated after the failure of CHT-RT for an advanced OTSCC extending to the base of tongue; the patient underwent close follow-up but experienced recurrent disease after 6 months; then, the patient underwent palliative CT and died of the disease 12 months after surgery.

Neck dissection yielded a mean number of lymph nodes of 53.1 (range of 0–139); in 77 cases (95.1%), the neck dissection yielded 18 or more lymph nodes, and in 4 cases (4.9%), <18 lymph nodes (3 of these patients were previously treated with neck dissection and underwent revision surgery for recurrent OTSCC).

Thirty-seven patients (45.7%) showed an upstage while shifting from a clinical to a pathological stage; according to the 8th AJCC staging system, 19 patients initially staged as cT1 resulted in 6 pT1 (31.6%), 8 pT2 (42.1%), and 5 pT3 (26.3%); 48 patients initially staged as cT2 resulted in 24 pT2 (50%), 22 pT3 (45.8%) and 2 pT4a (4.2%); 1 patient initially staged as cT3 and 13 patients initially staged as cT4a resulted in 1 pT3 (100%) and 13 pT4a (100%); 52 patients initially staged as cN0 resulted in 48 pN0 (92.3%) and 4 pN1 (7.7%); 11 patients initially staged as cN1 resulted in 9 pN1 (81.8%) and 2 pN2 (18.2%); and 18 patients initially staged as cN2 resulted in 16 pN2 (88.9%) and 2 pN3 (11.1%).

Thirty-nine patients (48.1%) showed upstaging while shifting from the 7th to the 8th AJCC staging system, and none of the patients were downstaged (see Table 4). A total of 19 patients initially staged as pT1 resulted in 6 pT1 (31.6%), 8 pT2 (42.1%) and 5 pT3 (26.3%); 45 patients initially staged as pT2 resulted in 23 pT2 (51.1%), and 22 pT3 (48.9%); 3 patients initially staged as pT3 resulted in 2 pT3 (66.7%) and 1 pT4a (33.3%); and 14 patients initially staged as pN1 resulted in 13 pN1 (92.9%) and 1 pN2a (7.1%). The histological identification of ENE was crucial; 6 patients initially staged as pN2c resulted in 4 pN2c (66.7%) and 2 pN3b ENE+ (33.3%).

**Table 4:** TNM staging system, AJCC 2010 and 2017, 7th and 8th edition.

**AJCC 2010**		**pN0**	**pN1**	**pN2a**	**pN2b**	**pN2c**	**pN3b**	**Total**
pT1	2010	17 (2)	2	0	0	0	0	19 (2)
	2017	6 (1)	0	0	0	0	0	6 (1)
pT2	2010	24 (3)	10	0	10	1	0	45 (3)
	2017	22 (3)	3	0	5	1	0	31 (3)
pT3	2010	1 (1)	0	0	1	1	0	3 (1)
	2017	14 (2)	8	1	6	0	0	29 (2)
pT4a	2010	6 (1)	2	1	1	4 (2)	0	14 (3)
	2017	6 (1)	2	1	1	3 (2)	2	15 (3)
Total	2010	48 (7)	14	1	12	6 (2)	0	81 (9)
	2017	48 (7)	13	2	12	4 (2)	2	

**Figure 2:**
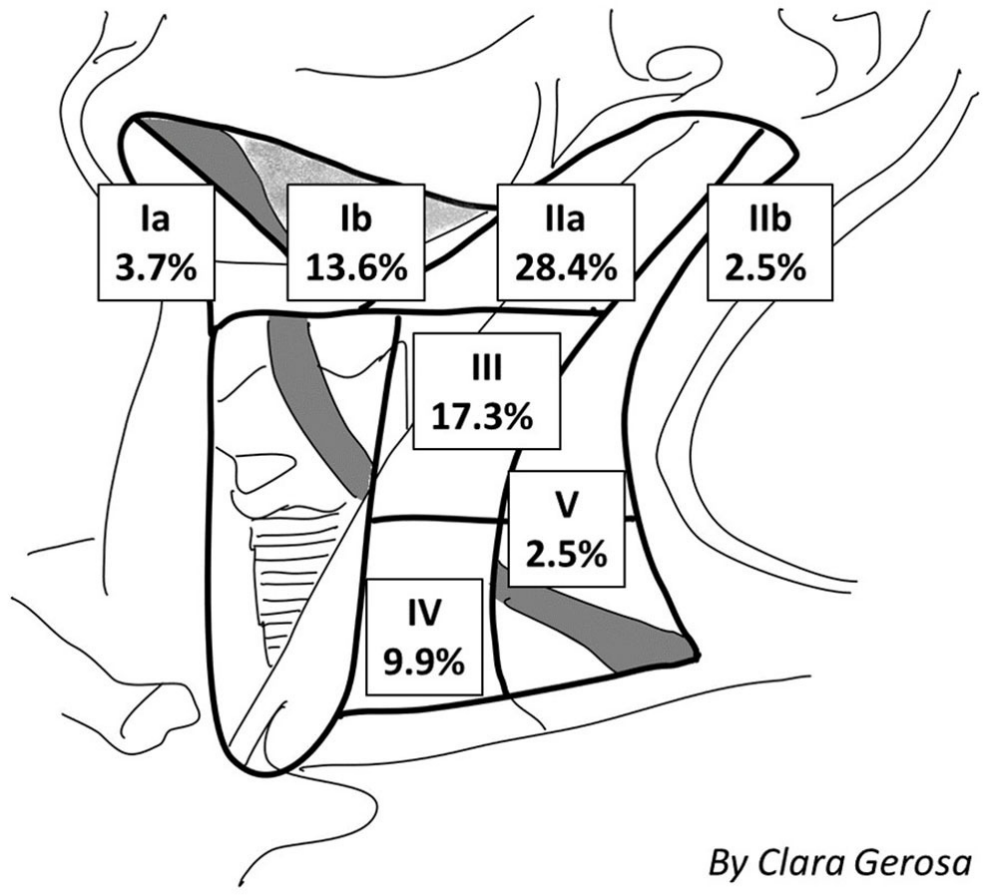
Percentage and site of incidence of nodal metastasis.

Definitive nodal involvement was confirmed in 33 patients (40.7%); in 27 patients (33.3%), it was ipsilateral, while in 6 patients (7.4%), it was bilateral or contralateral. In the majority of cases, lymph node involvement was localized: at the IIa cervical level in 23 patients (28.4%), the III level in 14 cases (17.3%), the Ib level in 11 cases (13.6%), the IV level in 8 cases (9.9%), the Ia level in 3 cases (3.7%), the llb level in 2 cases (2.5%), and the V level in 2 cases (2.5%), as shown in Figure 2. The mean LNR was 0.017; the LNR was 0 in 48 patients (59.3%), ≤ 0.09 in 30 patients (37%), and >0.09 in 3 patients (3.7%).

PNI was present in 9 cases (11.1%), LVI was present in 11 cases (13.6%), and concomitant PNI and LVI were present in 15 cases (18.5%). The absence of PNI or LVI was reported in 46 cases (56.8%). Only two lesions showed positive p16.

Twenty-two patients (27.1%) underwent adjuvant therapy (Table 5). Eleven patients (13.6%) underwent adjuvant RT, which was indicated on the basis of the advanced T stage (pT2 in 3 cases, 27.3%; pT3 in 7 cases, 63.6%; and pT4 in 1 case, 9.1%), pN+ (observed in 7 cases, 63.6%), PNI (observed in 9 cases, 81.8%), and LVI (observed in 4 cases, 36.4%). Nine patients (11.25%) underwent adjuvant CHT-RT, which was indicated on the basis of the advanced T stage (pT2 in 3 cases, 28.6%; pT3 in 4 cases, 42.8%; and pT4 in 2 cases, 28.6%), pN+ and LVI in 7 cases, LVI alone in 1 case, and pN+ alone in one case, while PNI was observed in 3 cases (42.8%). The mean RT dose was 58 Gy (range of 54–69.3 Gy) in 30–33 fractions (mean number of fractions was 31.1). Two patients previously treated by RT for other head and neck malignancy (2.5%) underwent adjuvant CHT alone, which was indicated on the basis of the advanced T stage (pT3 in 1 case, and pT4a in 1 case). Adjuvant therapy (RT alone, CHT alone or CHT-RT) was performed 8–16 weeks after surgery (mean time of 10 weeks) in 22 patients. In 8 patients, RT was not performed for different reasons despite being indicated: 2 patients refused RT, and 6 patients could not attend the RT sessions due to logistic/personal problems.

**Table 5:** Series of patients who underwent adjuvant therapy and who experienced relapse of disease.

**Patient**	**Previous therapies**	**Surgery**	**pTNM**	**PNI/LVI**	**Adjuvant therapy**	**Recurrence/Time of relapse (years)**	**rTNM**	**Salvage therapy**
1	–	Type IIIb glossectomy, SND, forearm free flap	pT2N0M0	LVI	CHT (Taxit + 63 Gy)	–	–	–
2	–	Type IIIb glossectomy, SND, forearm free flap	pT2N2bM0	LVI	–	Nodal relapse (contralateral lymph node)	rpTxN2cM0	SND
3	–	Type IIIb glossectomy, MRND, forearm free flap	pT3N2bM0	–	CHT (Al Sarraf + 63 Gy)	Nodal relapse (contralateral lymph node)	rypTxN3M0	SND + CHT (Taxit)
4	–	Type IIIb glossectomy, FND + SND, forearm free flap	pT4aN1M0	LVI	CHT-RT (Taxit + 63 Gy)	Nodal relapse (contralateral lymph node)	rypTxN3M0	RND
5	–	Type IIIb glossectomy, FND, forearm free flap	pT2N2bM0	LVI	CHT-RT (CDDP + 63 Gy)	–	–	–
6	Surgery + RT	Type IVb glossectomy, SND, VRAM free flap	rpT4aN0M0	–	CHT (Taxit)	Local (floor of mouth)	rycT4aN0M0	Palliative CHT
7	Surgery + RT	Type IIIb glossectomy, marginal mandibulectomy, DCIA free flap	rypT3N0M0	–	CHT (Taxit)	Local extended to pterygoid space	rycT4bN0M0	Palliative CHT
8	–	Type IVa glossectomy, FND + FND, forearm free flap	pT2N2cM0	–	CHT-RT (Taxit + 63 Gy)	–	–	–
9	–	Type IIIb glossectomy, SND, forearm free flap	pT3N2bM0	LVI	CHT-RT (Platin + 60 Gy)	–	–	–
10	CHT-RT	Type V glossectomy, SND + SND, VRAM free flap	ypT4aN0M0	PNI-LVI	–	Local (floor of mouth)	rycT4aN0M0	CHT
11	Surgery + RT	Type IIIb glossectomy, SND, forearm free flap	ypT2N0M0	–	–	Local (floor of mouth) Distant (lung)	rycT4aN0M1	Palliative CHT
12	–	Type V glossectomy, SND + SND, VRAM free flap	pT3N0M0	–	–	Nodal relapse (homolateral lymph node)	rpT0N2aM0 ENE+	SND + CHT-RT
13	–	Type IIIb glossectomy, FND, forearm free flap	pT3N2bM0	PNI-LVI	CHT-RT (Taxit + 59 Gy)	–	–	–
14	–	Type IIIb glossectomy, SND, forearm free flap	pT3N1M0	LVI	–	Local (base of tongue) Distant (lung)	rcT4aN2cM1	CHT
15	–	Type IIIb glossectomy, FND, forearm free flap	pT2N1M0	LVI	–	Local (floor of mouth) Distant (mediastinum)	rcT4aN2bM1	CHT
16	–	Type IIIb glossectomy, FND, forearm free flap	pT4aN2bM0	PNI-LVI	CHT-RT (Taxit + 63 Gy)	–	–	–
17	–	Type IVa glossectomy, FND + FND, forearm free flap	pT4aN3bM1 ENE+	PNI-LVI	–	Local (tongue) Distant (C1)	rcT4aN0M1	Palliative CHT
18	–	Type IIIb glossectomy, FND, forearm free flap	pT3N2bM0	PNI-LVI	RT (63 Gy)	Distant (lung, T11, L1)	rycT0N0M1	Palliative CHT
19	–	Type IIIb glossectomy, SND, forearm free flap	pT3N0M0	PNI	RT (63 Gy)	–	–	–
20	–	Type IIIb glossectomy, FND, forearm free flap	pT3N2bM0	PNI-LVI	CHT-RT (Taxit + 63 Gy)	Distant (lung)	rycT0N0M1	CHT
21	–	Type IIIb glossectomy, SND + SND, forearm free flap	pT2N0M0	PNI	RT (54 Gy)	Nodal relapse (contralateral lymph node)	rypTxN2cM0	SND
22	–	Type IIIb glossectomy, FND, forearm free flap	pT2N2bM0	PNI-LVI	RT (54 Gy)	–	–	–
23	–	Type IIIb glossectomy, SND, forearm free flap	pT3N1M0	PNI-LVI	RT (54 Gy)	Distant (supraclavicular fat)	rcT0N0pM1	MRND
24	–	Type IIIb glossectomy, SND + SND, forearm free flap	pT4aN0M0	–	RT (60 Gy)	–	–	–
25	–	Type IVa glossectomy, FND + SND, forearm free flap	pT4aN2cM0	–	–	Local (tongue) Distant (lung)	rcT4aN0M1	Palliative CHT
26	–	Type IIIb glossectomy, FND, forearm free flap	pT3N1M0	PNI	RT (63 Gy)	–	–	–
27	–	Type IIIb glossectomy, FND, forearm free flap	pT2N2bM0	PNI-LVI	RT (63 Gy)	–	–	–
28	CHT (TPF) + CHT-RT (Erbitux)	Total glossectomy, FND + FND, ALT free flap	ypT4aN2cM0	PNI-LVI	–	Locoregional (tongue, contralateral lymph node)	rycT4aN2cM0	CHT (CDDP + Erbitux)
29	–	Total glossectomy, FND + FND, ALT free flap	pT4aN3bM0 ENE+	PNI-LVI	–	Distant (brain)	rcT0N0M1	–
30	Surgery	Type IIIb glossectomy, SND, forearm free flap	(m)pT3N0M0	PNI	RT (60 Gy)	–	–	–
31	–	Type IVb glossectomy, FND + SND, ALT free flap	pT3N1M0	–	RT (63 Gy)	–	–	–
32	–	Type IVa glossectomy, SND + SND, forearm free flap	pT3N1M0	PNI	RT (63 Gy)	–	–	–

*Taxit, Taxotere; CDDP, Cisplatinum; Al Sarraf, Cisplatinum + 5-Fluorouracil*.

Mean time of follow-up was 3.3 years, median time was 1.8 years with a range of 6 months−7 years.

During the follow-up, 18 patients (22.3%) experienced recurrence of the disease (mean time of recurrence of 10.1 months): 3 patients (3.7%) showed local recurrence (mean time of recurrence of 12 months), 5 patients (6.2%) showed lymph node recurrence (mean time of recurrence of 12.4 months), 1 patient local and node recurrence (1.2%) after 8 months, 5 patients (6.2%) showed locoregional recurrence associated with distant metastases (mean time of recurrence of 7.8 months), and 4 patients (5%) experienced distant metastases alone (mean time of recurrence of 9.8 months) (Table 5). Of the patients who experienced relapse of the disease during the follow-up, 9 (50%) were upstaged according to the 8th Edition of the AJCC staging system (1 pT1 was restaged as pT2, 5 pT2 as pT3, 1 pT3 as pT4a, and 2 pN2c as pN3b ENE+).

The 5-years DSS, OS, ORFS, LRFS and DMFS were 73.2, 66.8, 62.6, 67.4, and 86%, respectively (Table 6). The survival rates and univariate and multivariate analyses based on the different clinical characteristics are reported in Tables 6–10.

**Table 6:** Univariate analysis of the survival rates according to pT based on the TNM staging systems (AJCC 2010 and 2017) in all patients, and in naive patients.

**Patients' groups**		**5-years DSS%**	**SE**	**5-years OS%**	**SE**	**5-years ORFS%**	**SE**	**5-years LRFS%**	**SE**	**5-years DMFS%**	**SE**
All patients (*n* = 80)		73.2	6.2	66.8	6.6	62.6	7.3	67.4	7.4	86	4.4
pT1	AJCC 2010	100	0	69.7	15.7	87.1	8.6	87.1	8.6	94.1	5.7
	AJCC 2017	100	0	83.3	15.2	100	0	100	0	100	0
pT2	AJCC 2010	78.4	7.4	74.5	7.6	65.3	9.7	74.2	9.5	84.2	6.6
	AJCC 2017	88.7	6.3	78.7	9	83.1	8	83.1	8	96.4	3.5
pT3	AJCC 2010	33.3	27.2	33.3	27.2	33.3	27.2	33.3	27.2	66.7	27.2
	AJCC 2017	71	12.1	68.5	11.9	48.3	13.4	58.2	14.8	74.3	10.2
pT4a	AJCC 2010	33.9	16.8	33.8	16.8	33.8	16.8	36.9	18.1	84.5	11.3
	AJCC 2017	31.2	15.7	31.2	15.7	31.2	15.7	33.8	16.8	75.5	12.3
Naive patients (*n* = 71)		80.6	11	72.2	6.7	60.1	7.3	74	9.5	84.2	6.5
pT1	AJCC 2010	100	0	77.8	15.2	85.1	9.7	85.1	9.7	93.3	6.4
	AJCC 2017	100	0	40	29.7	100	0	100	0	100	0
pT2	AJCC 2010	81.8	6.8	77.6	7.1	67.9	9.7	77.4	9.3	83.6	6.9
	AJCC 2017	92.6	5.1	88.7	6.1	86.4	7.6	86.4	7.6	96	3.9
pT3	AJCC 2010	50	35.4	50	35.4	50	35.4	50	35.4	50	35.4
	AJCC 2017	80.7	8.7	77.7	8.9	55.3	12.7	67.4	13.3	72.8	10.7
pT4	AJCC 2010	53	18.7	53	18.7	53	18.7	58.3	19.8	80.8	12.3
	AJCC 2017	48.1	17.6	48.1	17.6	48.1	17.6	52.5	18.7	73.3	13.2
Patients previously treated by RT (*n* = 9)		0	0	0	0	0	0	0	0	–	–

*DSS, Disease-specific Survival; OS, Overall Survival; ORFS, Overall Relapse-free Survival; LRFS, Local Relapse-free Survival; DMFS, Distant Metastasis-free Survival*.

Patients with a LNR > 0.09 experienced significantly worse outcomes than patients with a LNR lower than 0.09 (Table 7).

**Table 7:** Univariate analysis of the survival rates according to histological risk factors.

**Patients' groups**		**5-years DSS%**	***p***	**5-years OS%**	***p***	**5-years ORFS%**	***p***	**5-years LRFS%**	***p***	**5-years DMFS%**	***p***
		**SE**		**SE**		**SE**		**SE**		**SE**	
Previous RT	Naive patients	80.6	**0.011**	72.2	**0.006**	60.1	**0.049**	74	**0.01**	84.2	–
		11		6.7		7.3		9.5		6.5	
	Previous RT	0		0		0		0		–	
		0		0		0		0			
Stage AJCC 2017	All patients										
	I	100	**0.0002**	42	**0.002**	100	**0.003**	100	**0.02**	100	0.07
		0		30.4		0		0		0	
	II	93.8		80.4		86.5		86.5		100	
		6.1		10.7		8.9		8.9		0	
	III	79.5		79.5		51.3		55.6		77.9	
		11.9		11.9		15.2		15.9		10	
	IV	42.9		41.2		42.9		50.7		76.1	
		11.9		11.5		11.9		13.1		9.4	
	Naive patients										
	I	100	**0.0031**	40	**0.0135**	100	**0.0383**	100	0.1494	100	0.1109
		0		29.7		0		0		0	
	II	100		94.7		91.7		91.7		100	
		0		5.1		7.8		8		0	
	III	89.9		89.9		56		61.6		75	
		6.7		6.8		15.5		16		11.2	
	IV	55.9		53.6		55.9		66.1		76.1	
		11.7		11.4		11.7		12.2		9.4	
pN	All patients										
	0	86.9	**0.0001**	72.5	**0.0001**	72.4	**0.0001**	72.4	**0.0001**	97.2	**0.0001**
		6.6		9.1		9.3		9.3		2.7	
	1	53		53		53		53		63.6	
		23.3		23.3		23.3		23.3		17.7	
	2	52.8		49.9		63.3		63.3		76.9	
		13.2		12.8		13.6		13.6		11.7	
	3	0		0		0		0		0	
		0		0		0		0		0	
	Naive patients										
	0	97.5	**<0.0001**	86	**<0.0001**	81.2	**<0.0001**	81.2	**<0.0001**	96.9	**<0.0001**
		2.5		7.2		8.4		8.4		3.1	
	2	53		53		42.4		53		63.6	
		23.3		23.3		21		23.3		17.7	
	3	61.2		57.4		61.3		74.9		75	
		13.8		13.4		13.8		13		12.5	
	4	0		0		0		0		0	
		0		0		0		0		0	
LNR	All patients										
	0	92.1	**<0.0001**	76.5	**<0.0001**	74.5	**<0.0001**	74.5	**<0.0004**	97.1	**<0.0001**
		4.4		8.6		8.8		8.8		2.8	
	≤ 0.09	54		52.2		49		61		70.1	
		11.6		11.3		11.5		12		10.6	
	>0.09	0		0		0		0		33.3	
		0		0		0		0		27.2	
	Naive patients										
	0	97.4	**<0.0001**	85.9	**<0.0001**	81.1	**<0.0001**	81.1	**<0.0001**	96.9	**<0.0001**
		2.5		7.2		8.4		8.4		3	
	≤0.09	63		60.7		57.2		72		68	
		12		11.8		12.2		12		11	
	>0.09	0		0		0		0		33.3	
		0		0		0		0		27.2	
Grading	All patients										
	1	80.5	0.6	68.6	0.8	80.5	0.1	80.5	0.3	96.3	0.14
		7.9		9.4		7.9		7.9		3.6	
	2	60.1		58.5		37.8		44.1		75.2	
		13.1		12.9		14.2		16.1		8.5	
	3	83.3		55.6		69.4		76.4		90.1	
		10.8		23.8		15.5		15.5		8.7	
	Naive patients										
	1	82.3	0.8265	74.8	0.9927	82.3	0.2765	82.3	0.5431	95.8	0.1543
		8.2		8.9		8.2		8.2		4.1	
	2	78.6		76.4		52.6		61.7		73.5	
		7.9		8		14		15.4		8.9	
	3	90.9		90.9		75.8		83.3		90.9	
		8.7		8.7		15.6		15.2		8.7	
PNI	All patients										
	Absent	78.7	0.07	68	0.08	71.6	**0.006**	71.6	0.17	91.5	0.53
		6.6		7.8		7.9		7.9		4.1	
	Present	63.1		60.5		43.8		59		72.3	
		11.5		11.3		12.8		14.4		11	
	Naive patients										
	Absent	83.9	0.3313	75.8	0.2963	76.6	**0.0271**	76.6	0.6917	90.9	**0.0285**
		5.7		7		7.4		7.4		4.4	
	Present	76.4		72.6		49.3		70.3		67.2	
		10.4		10.6		15		17		12.7	
LVI	All patients										
	Absent	84.5	**0.001**	71.9	**0.004**	73.5	**0.0008**	73.5%	**0.04**	98%	**0.0001**
		6.2		8.2		8.6		8.6		2	
	Present	48.3		46.5		38.7		51.9		59.1	
		12.7		12.3		11.9		13.7		11.6	
	Naive patients										
	Absent	91	**0.0028**	81.3%	**0.008**	79.5%	**0.0019**	79.5%	0.1241	97.8%	**<0.0001**
		4.3		6.9		7.8		7.8		2.2	
	Present	53.4		50.8		41.5		59		52.2	
		14.9		14.4		13.8		16.2		12.7	
PNI-LVI	All patients										
	Absent	82.4	**0.002**	69.3	**0.002**	76.1	**0.0013**	76.1	0.06	97.5	**0.0006**
		6.8		8.6		8.8		8.8		2.5	
	Both present	42.7		39.9		32.1		57.9		53.7	
		14.8		14.1		14.4		17.2		16.2	
	Naive patients										
	Absent	85.9	**0.0081**	78.1%	**0.0036**	74.7%	**0.0033**	74.7%	0.9396	92.9%	**<0.0001**
		5.1		16.6		7.2		7.2		3.8	
	Both present	56.3		51.1		37.5		87.5		37.5	
		16.5		15.8		18.9		11.7		18.9	

*DSS, Disease-specific Survival; LRFS, Local Relapse-free Survival; ORFS, Overall Relapse-free Survival; DMFS, Distant Metastasis-free Survival; OS, Overall Survival; PNI, Perineural Invasion; LVI, Lymphovascular Invasion; LNR, Lymph node ratio*.

Univariate analysis showed that patients with previous RT, stage IV disease, nodal involvement, and LVI had significantly worse survival rates (Table 7).

Multivariate analysis focused to naive patients (*n* = 71) showed that LVI, LVI-PNI (Table 8), advanced stage of disease (Tables 8, 9), and node involvement (Table 10) resulted as reliable prognostic factors, and patients with the same tumor stage and histological risk factors who did not undergo adjuvant therapy experienced significantly worse outcomes.

**Table 8:** Multivariate analysis of ORFS, LRSF, and DMFS rates according to stage and histological risk factors in naive patients.

**Variables**		**5-years ORFS%**	**SE**	***p***	**5-years LRFS%**	**SE**	***p***	**5-years DMFS%**	**SE**	***p***
Stage I–II	**PNI-LVI**			**0.004**	**PNI-LVI**		0.1038	**PNI-LVI**		**0.0002**
	Absent	100	0		100	0		100	0	
	Present	75	21.7		75	21.7		100	0	
Stage III–IV	**PNI-LVI**				**PNI-LVI**			**PNI-LVI**		
	Absent	**69.3**	10.3		62.3	10.3		**86.2**	6.5	
	Present	37.5	18.9		87.5	11.7		37.5	18.9	
**Variables**		**5-years ORFS%**	**SE**	***p***	**5-years LRFS%**	**SE**	***p***	**5-years DMFS%**	**SE**	***p***
Stage I–II	**LVI**			**0.0008**	**LVI**		0.074	**LVI**		**<0.0001**
	Absent	92.9	6.9		92.9	6.9		100	0	
	Present	100	0		100	0		100	0	
Stage III–IV	**LVI**				**LVI**			**LVI**		
	Absent	**70.1**	11.7		70.1	11.7		**96**	3.9	
	Present	32.8	14		51.1	18.1		43.7	13.7	

*ORFS, Overall Relapse-free Survival; LRFS, Local Relapse-free Survival; DMFS, Distant Metastasis-free Survival; PNI, Perineural Invasion; LVI, Lymphovascular Invasion*.

Table 9Multivariate analysis of the survival rates of III-IV stage naive patients according to histological risk factors and adjuvant treatment.

**Table:** 

**Patients' groups**		**5-years DSS%**	**SE**	***p***	**5-years OS%**	**SE**	***p***
Stage III–IV	**LVI absent**			**0.0008**	**LVI absent**		**0.0008**
	**CHRT/RT**				**CHRT/RT**		
	No	94.8	5.5		82.8	7.6	
	Yes	70	18.2		70	18.2	
	**LVI present**				**LVI present**		
	**CHRT/RT**				**CHRT/RT**		
	No	47.6	17.4		42.9	17.4	
	Yes	65.5	17.3		65.6	17.3

**Table:** 

**Patients' groups**		**5-years ORFS%**	**SE**	***p***	**5-years LRFS%**	**SE**	***p***	**5-years DMRFS%**	**SE**	***p***
Stage III–IV	**LVI absent**			**0.0001**	**LVI absent**		**0.0001**	**LVI absent**		**0.0001**
	**CHRT/RT**				**CHRT/RT**			**CHRT/RT**		
	No	86.8	8.3		86.8	8.3		97.2	2.7	
	Yes	40	20.3		40	20.3		100	0	
	**LVI present**				**LVI present**			**LVI present**		
	**CHRT/RT**				**CHRT/RT**			**CHRT/RT**		
	No	23.8	19.3		26.8	21.4		23.8	19.3	
	Yes	56.1	17.2		80	17.9		70.1	14.7	

*DSS, Disease-specific Survival; OS, Overall Survival; LRFS, Local Relapse-free Survival; ORFS, Overall Relapse-free Survival; DMFS, Distant Metastasis-free Survival; LVI, Lymphovascular Invasion; CHRT/RT, Chemoradiotherapy/Radiotherapy*.

Table 10Multivariate analysis of the survival rates in pN+ naive patients according to histological risk factors and adjuvant therapy.

**Table:** 

**Variables**		**5-years DSS%**	**SE**	***p***	**5-years OS%**	**SE**	***p***
pN+	**LVI absent**			**0.0008**	**LVI absent**		**0.0002**
	**CHRT/RT**				**CHRT/RT**		
	No	61.7	18		61.7	18	
	Yes	100	0		100	0	
	**LVI present**				**LVI present**		
	**CHRT/RT**				**CHRT/RT**		
	No	0	0		0	0	
	Yes	60	19.7		60	19.7

**Table:** 

**Variables**		**5-years ORFS%**	**SE**	***p***	**5-years LRFS%**	**SE**	***p***	**5-years DMRFS%**	**SE**	***p***
pN+	**LVI absent**			**0.0009**	**LVI absent**		**0.0035**	**LVI absent**		**<0.0001**
	**CHRT/RT**				**CHRT/RT**			**CHRT/RT**		
	No	61.7	18		61.7	18		85.7	13.2	
	Yes	100	0		100	0		100	0	
	**LVI present**				**LVI present**			**LVI present**		
	**CHRT/RT**				**CHRT/RT**			**CHRT/RT**		
	No	0	0		0	0		0	0	
	Yes	50	18.8		75	21.7		66.7	16.1	

*DSS, Disease-specific Survival; LRFS, Local Relapse-free Survival; ORFS, Overall Relapse-free Survival; DMRFS, Distant Metastasis-free Survival; OS, Overall Survival; LVI, Lymphovascular Invasion; CHRT/RT, Chemoradiotherapy/Radiotherapy*.

## Discussion

**Figure 3:**
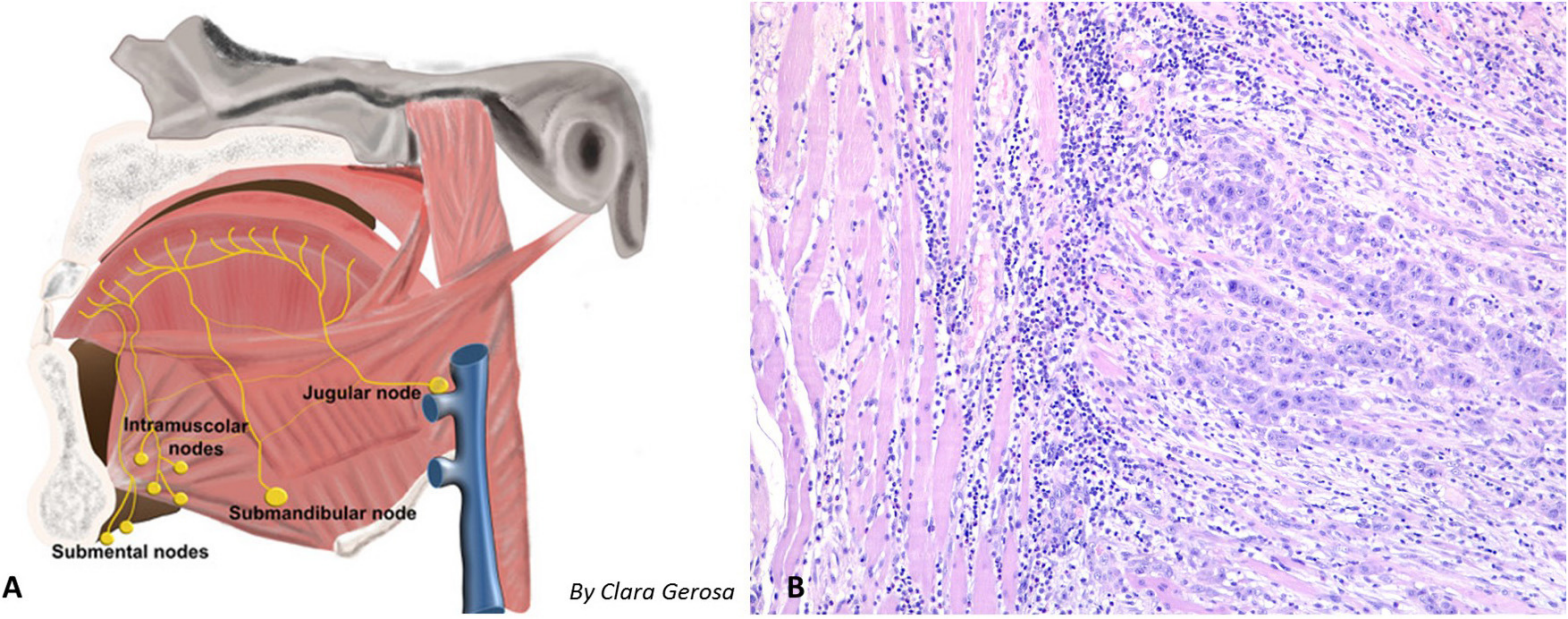
**(A)** Shema of the locoregional lymphatic spread of tongue malignancy, with possible intramuscular metastasis. **(B)** 10× Histology of an intramuscular metastasis (geniohyoid muscle) observed in a patient with pT3N2a squamous cell carcinoma of the tongue; the muscle fibers dissociated by a proliferation of epithelioid cells organized in solid nests with infiltrative growth pattern. The epitelioid cells showed polimorphic and polidimensional nuclei with prominent nucleoli, atypical mitosis and wide eosinophilic cytoplasms. In the background around and inside the tumor there is an inflammatory response composed by lymphocytes and monocytes.

The treatment of OTSCC remains a major therapeutic challenge, and surgery plays a fundamental role in achieving locoregional control (27–29) with the main goal of removing the primary tumor with adequate margins of healthy tissue; however, the definition of an “acceptable free margin” is essentially unclear (6, 30–32). Calabrese et al. (33) showed that DSS and OS improved in patients with advanced-stage OTSCC after CTS because of a tridimensional control of the superficial and deep extension of the tumor; as a consequence, CTS can be considered a sound oncologic option and could be applied routinely with the aid of primary microvascular free flap reconstruction, which has replaced, in the majority of cases, the use of loco-regional flaps. After wide resection, a reconstructive procedure is needed to fill the anatomical defect, reduce the risk of post-operative complications (such as salivary fistula), and recreate a functional volume, thereby improving residual tongue movements and functions (34). Obtaining healthy vascularized tissue from the donor site protects the mandible, thereby reducing eventual radio-induced complications (35). In our series, no fistula or radionecrosis occurred after RT; indeed, radionecrosis and fistula were present before CTS in two cases previously treated elsewhere with local resection without reconstruction followed by RT, which recovered after our microvascular reconstruction. The CTS approach associated with microvascular reconstruction has been adopted routinely in our department since November 2010 and allowed the resection on healthy tissue in 98.8% of patients despite the pT stage; a single close margin was observed in one patient. This surgical approach also allows the radical removal of microscopic peritumoral buds and all the lymphatics within the anatomical compartment where the tumor can develop (Figures 3A,B). Although this surgical strategy is technically more complex than the classic “wide resection” approach, which includes a transtumoral approach (36) without reconstruction, in our series, CTS was not burdened by a higher incidence of major complications than has been reported in the literature (33, 36): we observed one post-operative death (1.2% of all patients; an elderly patient whose AACCI score was 7 and who died of heart failure the day after the surgical procedure), and no patient experienced flap failure. The additional operating time was defined as the time required to perform the microanastomosis since the harvesting of the flap was contemporary to the resection, and the suturing of the flap was performed during the time spent waiting for frozen sections.

The literature review showed that patients with OTSCC have a 5-years DSS, OS and ORFS from 51.1 to 77.8%, 31.5 to 70.7%, and 50 to 68.1% respectively (6, 37–39); our results are comparable with the best reported in the literature (Table 6). The OS observed in our series could be related to the high incidence of comorbidities: only 31 patients did not have any comorbidities (38.3%), while 27 patients had 1 or 2 comorbidities (33.3%), and 23 patients had ≥3 comorbidities (28.4%) since the last condition was not considered an absolute contraindication to CTS. Although CTS followed by microvascular reconstruction could be considered more aggressive than excision, it produces better loco-regional control than more limited resections. Sinha et al. (36) treated their T1 and T2 patients by multiblock transoral resection with a 1 cm free margin evaluated intraoperatively with the operative microscope, achieving the following oncologic results: 5-years DSS, ORFS and OS: 88.6, 70, and 78%, respectively, in T1 patients and 74.4, 56.8, and 60.2%, respectively, in T2 patients. Five-years DSS, OS, and RFS rates of T1 and T2 lesions reported in literature in patients are 91.7–95, 75–84, 71.9–73% and 79.3–92, 59–73.3, 66.9–80%, respectively (40, 41). These results seem lower than those achieved in our study (Table 6) and in the experience of Calabrese et al. (33).

After CTS, 28% of patients in the study of Calabrese et al. (6) and 22.3% of our patients experienced recurrent disease; in our series, relapse of the disease was observed in 2 patients with stage II disease (2.5%), in 5 patients with stage III disease (6.2%) and 11 patients with stage IV disease (13.6%).

Recurrence, which can occur despite radical histologically-proven resection, remains a challenge to improving understanding of OTSCC biology. These neoplasms do not always show the same biological behavior, and different clinical risk factors may be associated with a higher aggressiveness of the tumor and could require different therapeutic strategies.

An association between HPV and oral cavity cancer has been described in the literature (42). In the present series, only 2 lesions were p16-positive, and the virus genome was detected in only 1 patient with OTSCC. However, in this patient, the lesion showed only the focal expression of p16 and was not considered an HPV-related disease.

Xu et al. (43) demonstrated that when ECE-1 is overexpressed in head and neck squamous cell carcinoma (HNSCC), poor tumor differentiation is associated with worse prognosis; however, in our series, patients with poorly differentiated disease did not experience significantly worse survival rates than patients with well-differentiated lesions (Table 7).

Recurrence after previous treatment was associated with worse local control: of the 71 naive patients, 16 (22.5%) experienced relapse of the disease, while of the 9 patients with previous RT for head and neck malignancies, 5 (55.6%) relapsed, *p* = 0.034.

Advanced T stage, nodal involvement, ENE, and poorer differentiation are well-known prognostic factors for OTSCC (44), and the latest edition of the AJCC TNM staging system has been changed on the basis of DOI and ENE (8, 9). The evaluation of the tumor's thickness was defined by Moore et al. (45) as the deepest point of tumor invasion (from the mucosal surface) in the tissue. The tumor's thickness is currently expressed by the DOI at histology, defined as the distance from the level of the basement membrane of the closest adjacent normal mucosa (22). DOI is considered a main prognostic factor associated with the risk of lymph node involvement (46–49). The significance of the DOI in the TNM staging system has been recently validated in several studies. Lydiatt et al. (8). evaluated a large population of 1,788 patients and confirmed that the DOI is a significant prognostic factor for the prediction of DSS and OS. Matos et al. (38) observed that both RFS and OS were significantly lower in patients undergoing upstaging after the application of the 8th edition of the TNM staging system (*p* = 0.007 and *p* = 0.017, respectively). Tirelli et al. (39) observed a major correlation between increased pT categories and DSS (*p* = 0.01) using the 8th edition of the TNM staging system, concluding that DOI > 10 mm is an independent prognostic factor that significantly impacts DSS (*p* = 0.001). In our series, 39 lesions (48.1%) were upstaged after restaging with the 8th edition of the TNM staging system, and in accordance to the recent literature, DSS decreases progressively according to the increasing pT category. The DSS rates based on the 7th and 8th editions were as follows: pT1 100 vs. 100%, pT2 78.4 vs. 88.7%, pT3 33.3 vs. 71%, and pT4 33.9 vs. 31.2% (Table 6), confirming that as found in the present study, the recent revision of the TNM staging system improves the correlation between T stage and prognosis and allows a better classification of OTSCC patients.

The involved margins, close margins, tumor's size and the depth of invasion of the extrinsic muscles are considered as negative prognostic factors (50, 51) and, in some cT2 tumors, it is difficult to determine before surgery whether or not the extrinsic lingual muscles are involved (51). The CTS approach allows for a resection performed along anatomic boundaries to the neoplastic spread with a complete resection even when the lesion present insidious paths of spread. Despite the three-dimensional extension of the disease, CTS, in the present series, seemed to ensure that free margins were achieved in all cases and had a positive impact on locoregional control of the disease; the neoplastic spread routes were removed *en bloc*.

The restaging of all our patients according to the 8th edition of the TNM staging system confirmed that CTS was not an overtreatment in patients previously staged as pT1 according to the 7th edition of the TNM staging system: 29 patients (35.8%) showed an upstage while shifting from a clinical T1-T2 to a T3-T4 pathological stage according to the 8th AJCC staging system and, among them, 5 were initially staged as cT1 and 24 were cT2; in these patients, a different surgical approach could have been associated with incomplete resections that were never observed in the present series. Furthermore, before 2017, the choice of the CTS approach resulted in the removal of the T-N tract potentially affected by satellite lesions or micrometastases in 27 patients (33.3%) who showed upstaging from pT1-pT2 to pT3-pT4 while shifting from the 7th to the 8th AJCC staging system; if we had performed a transoral resection in this class of tumors, we would probably have obtained worse prognoses.

Tagliabue et al. showed that on 95 patients classified as pT1-3 only 6 patients had the T-N tract involved while in 138 classified as pT4, 31 patients had a positive T-N tract (52); these findings could justify a “tailored” less aggressive resection (i.e., transoral laser resection without CTS) in case of cT1 lesions of the anterior tongue and without preoperative signs at MRI and or CT of involvement of the “anatomical barriers” for neoplastic spread. Less aggressive excisions could also be considered on the basis of age, comorbidities, previous treatments, immunological status, and patient's choice.

In the study by Tirelli et al. (39) and in our series, the new pT classification showed a better correlation with survival and oncologic outcomes. We observed that pT1–pT3 lesions according to the 8th AJCC staging system showed a significantly better prognosis than pT4 lesions; the worse prognosis of more extended lesions does not seem to be strongly related to the surgical procedure (histology confirmed free margins of resection in all advanced cases), and recurrences may be due to the insidious ways of diffusion that cannot be controlled through surgery alone.

In our series, the recent revision of the TNM staging system led to an upstaging of the pN in three patients (3.7% of the whole series). The main changes were due to the status of the pathological ENE, defined as the extension of metastatic carcinoma from the lymph node outside the nodal capsule (8). The presence of the stromal inflammatory reaction has been considered in the recent TNM staging system as an independent and reliable prognostic factor (8, 9, 33). The poor prognosis of patients with pathological ENE has been evaluated in our series: ENE was confirmed in 3 cases (1 pN2a and 2 pN3b), and after a 3-years follow-up, two patients died from the disease, whereas one patient died from stroke. Univariate analysis showed that pathological ENE was the worst histological prognostic factor and was associated with significantly worse oncologic outcomes (Table 7).

PNI, LVI and LNR are clear signs of an increased “imbalance” between oncogenes and tumor suppressor genes that promotes the neoplastic spread showing aggressiveness of the tumor also if they are not at the margin of the resection. It is hypothesized a morph-functional sequence when defining the steps of the metastatic cascade that includes the promotion of tumor neo-angiogenesis, synthesis of proteinases that helps cell intra and extravasation, synergism between altered adhesion molecules and proteinases, and loss of local immune-surveillance (53).

Many authors have associated PNI with local recurrence and lower OS (54–58) and have also observed that adjuvant therapy can have a significant positive impact on survival rates (*p* = 0.022). In our series, PNI did not significantly impact locoregional control or survival rates (Table 7). These data can be explained in our cases because PNI, although it is an indicator of histological aggressiveness, it was observed within the tumor but always far from the surgical margins, supporting the application of CTS, that allows wide resections, minimizing the negative impact of the presence of PNI on prognosis. Conversely, the presence of PNI in the surgical margins or the histological finding of cranial nerve invasion [defined as *perineural spread* by Brown (59)] could play a strong negative prognostic role, but it was never observed in our series. The impact of LVI on locoregional control and recurrence and survival rates has been widely demonstrated in other malignancies, such as hypopharyngeal and esophageal carcinoma, in which LVI is an independent prognostic factor (60, 61). In a recent study, Fives et al. (62) showed that the DOI (*p* = 0.009), LVI (*p* = 0.006), PNI (0.003) and nodal metastases (*p* = 0.02) had a significant negative impact on OS, and after the multivariate analysis, only LVI was associated with significantly worse OS (*p* = 0.009). In a cohort of 289 patients with OSCC, Quinlan-Davidson et al. (63) observed that LVI was associated with nodal involvement (*p* = 0.01) and DOI > 1.5 cm (*p* = 0.003) and suggested that it could be considered an independent negative prognostic factor (*p* = 0.006). Cassidy et al. (64) reported that the presence of LVI in patients without nodal involvement (N0) is associated with worse local control (*p* < 0.01), worse locoregional control (*p* < 0.01) and a lower OS (*p* = 0.01); consequently, it should be considered an indication for adjuvant therapy. In our series, univariate analysis showed that LVI is a prognostic factor associated with a significantly worse ORFS (*p* = 0.0025) and DMFS (*p* = 0.0006). Compared with the findings of Chen et al. (65) the prognostic value of LVI was more evident in our patients with stage III-IV disease (ORFS: *p* = 0.002; DMFS: *p* = 0.0014), especially when associated with PNI (Table 8). Multivariate analysis confirmed that patients with stages III-IV disease, node involvement and LVI experienced significantly worse outcomes especially when they refused adjuvant CHT-RT (Tables 9, 10).

In the literature, the LNR has been considered an additional factor for estimating prognosis (66, 67). In our series, we considered the cut-off value of 0.09 on the basis of the meta-analysis of Talmi et al. (22) who identified 28 studies in the literature that addressed the prognostic value of the LNR and reported a range of cut-off values of the LNR associated with prognosis between 0.02 and 0.20, with an average of 0.09. In our series, patients with an LNR higher than 0.09 experienced significantly worse outcomes. In these patients the higher LNR was due to the high number of metastatic nodes since the neck dissections yielded a mean number of lymph nodes of 53.1 (range of 18–134 nodes removed). The systematic use of this parameter should be associated with high-quality neck dissection (number of lymph nodes removed per level) to avoid statistical bias due to limited neck dissections.

AHNS guidelines (24) support adjuvant therapy in high-risk OTSCC (advanced stages, multiple nodal involvement, ENE+, positive margins) and for intermediate-risk OTSCC only when one or more negative prognostic factors, such as LVI or multiple nodal involvement, are observed; adjuvant therapy is also indicated in specific situations, such as early-stage OSCC with positive margins, which otherwise has a negative prognosis (68). The appropriateness of this multidisciplinary management was underlined in our series: adjuvant therapy showed a significant positive role in improving the prognosis of patients with stage III and IV disease associated with LVI, while patients with early-stage disease or without LVI did not experience a significant benefit from adjuvant therapies (Table 8).

In the majority of the patients, the five quality metric criteria according to Cramer et al. (25) were met, although eight patients did not undergo RT despite indications due to personal choice or logistic/personal problems that could not be overcome by patients or relatives. DSS, OS, and ORFS were significantly worse in these patients who did not undergo adjuvant RT than in patients who underwent RT (Tables 9, 10).

Functional outcomes after CTS showed a high recovery rate of adequate chewing and swallowing functions (97.5%). Complete removal of the extrinsic muscles from their bony insertions does not increase the functional defect any more than partial removal of the muscle involved (51). In our patients, the free flap allowed for a complete closure of the oral pelvis and an adequate volume of the reconstructed tongue, facilitating the oral phase of swallowing. In our series, all but two patients could be discharged from the hospital without a nasogastric feeding tube. Type IV and V glossectomies, although rarely performed, was burdened by higher disfunction, needing in two cases compensatory PEG.

In conclusion, our study pointed out that CTS was associated with a high rate of tumor-free margins in all stages of OTSCC. Oncologic results obtained with CTS were better than those obtained with traditional transoral or multiblock resections. Immediate free flap reconstruction was not burdened by major complications. Adequate or normal function was regained in all but two patients. Adjuvant therapy was indicated in patients with advanced disease and negative prognostic factors according to AJCC 2017 and was not burdened by complications probably due to the presence of a well-vascularized transplanted tissue.

## Data Availability Statement

All datasets generated for this study are included in the article/Supplementary Material.

## Ethics Statement

The studies involving human participants were reviewed and approved by Comitato Etico dell'Azienda Ospedaliero-Universitaria di Cagliari. The patients/participants provided their written informed consent to participate in this study.

## Author Contributions

FC, DQ, and RP designed the study, analyzed the data, and wrote the manuscript. EG, CM, VM, and MT collected and analyzed the data. CG and JZ performed the experiments. NC and AF edited the manuscript. All authors contributed to the article and approved the submitted version.

## Conflict of Interest

The authors declare that the research was conducted in the absence of any commercial or financial relationships that could be construed as a potential conflict of interest.
