# Supplementary material for: Compartmental Surgery With Microvascular Free Flap Reconstruction in Patients With T1–T4 Squamous Cell Carcinoma of the Tongue: Analysis of Risk Factors, and Prognostic Value of the 8th Edition AJCC TNM Staging System
Source: Front Oncol. 2020 Jul 14;10:984. doi: 10.3389/fonc.2020.00984 (PMC7372302; doi:10.3389/fonc.2020.00984)
Supplement: Supplementary file 1 [file Data_Sheet_1.PDF]

Number	Patient	Internal Number	Day of Birth	Month of Birth	Year of Birth
1	LV	1079/2010	11	9	1951
2	PF	163/2011	19	12	1942
3	FP	324/2011	10	11	1964
4	ZR	101/2011	30	7	1968
5	LL	223/2011	14	12	1951
6	AE	*02/2013	3	2	1968
7	SR	955/2011	29	3	1968
8	OG	977/2011	1	8	1942
9	PI	540/2014	8	2	1958
10	PE	1125/2011	5	11	1938
11	LM	53/2013	15	12	1931
12	GPP	18/2012	21	8	1945
13	SA	85/2012	1	4	1948
14	MG	539/2012	11	1	1953
15	SGP	193/2012	21	8	1948
16	DS	323/2012	26	10	1946
17	SGP	48/2018	12	9	1954
18	PG	365/2014	18	4	1947
19	FR	1081/2012	29	6	1963
20	ML	468/2012	1	8	1947
21	MV	583/2012	8	6	1956
22	CB	749/2012	8	12	1945
23	SC	822/2012	28	3	1958
24	CP	912/2013	12	8	1949
25	SG	966/2012	13	7	1957
26	CM	996/2012	1	10	1961
27	CG	1079/2012	6	6	1961
28	LC	61/2013	23	6	1952
29	LL	85/2013	8	5	1957
30	AM	567/2013	22	10	1971
31	DGG	1067/2013	26	4	1980
32	SG	1100/2013	3	1	1951
33	MA	1128/2013	16	3	1986
34	FG	269/2014	12	10	1964
35	FM	298/2014	12	7	1958
36	MRR	470/2014	21	8	1953
37	LL	736/2014	14	12	1951
38	PM	769/2014	13	7	1957
39	PG	928/14	12	3	1981
40	CB	435/2015	7	1	1957
41	MFM	1197/2014	8	12	1962
42	MBL	1172/14	1	1	1940
43	SAG	1394/2014	23	4	1956
44	PGI	45/2015	22	6	1969
45	FM	180/2015	1	2	1939
46	GDS	228/15	1	6	1953
47	GAF	377/2015	25	7	1954
48	LA	794/2015	30	5	1939
49	SI	677/2015	29	3	1953
50	SR	714/2015	18	9	1952
51	ST	940/2015	25	4	1950
52	CA	1072/2015	19	11	1971
53	ML	1099/2015	3	1	1955

Birth	Age	Age	Sex (M=(	Preoperative Smoking (0=	Preoperative Alcohol (0=
1951,694064	59,18310502	59	0	1	1
1942,965982	67,96712329	67	0	1	1
1964,857991	46,18584475	46	0	1	1
1968,579452	42,50273973	42	1	1	0
1951,952283	59,53835616	59	0	1	1
1968,088813	43,5739726	43	0	0	0
1968,243379	43,55045662	43	0	1	1
1942,583333	69,23242009	69	0	1	1
1958,102511	53,82511416	53	0	1	1
1938,844292	73,11073059	73	0	0	1
1931,955023	80,01643836	80	1	0	0
1945,638128	66,38652968	66	0	1	0
1948,25	63,83219178	63	0	1	1
1953,02739726	59,07237443	59	0	1	1
1948,638128	63,54223744	63	0	1	1
1946,818493	65,46164384	65	0	2	0
1954,696804	57,59703196	57	0	1	1
1947,296575	65,06141553	65	0	1	1
1963,493379	48,91666667	48	0	2	0
1947,583333	64,84429224	64	1	0	0
1956,435845	56,08881279	56	0	1	1
1945,935845	66,7390411	66	0	1	1
1958,240639	54,49178082	54	0	1	1
1949,61347	63,19680365	63	0	1	1
1957,532877	55,3196347	55	0	1	1
1961,75	51,1216895	51	0	2	1
1961,430365	51,51917808	51	1	0	0
1952,476941	60,56689498	60	0	2	1
1957,352511	55,71050228	55	0	1	2
1971,807534	41,64200913	41	0	0	1
1980,318493	33,52579909	33	0	0	1
1951,005479	62,85799087	62	0	2	2
1986,207763	27,67488584	27	0	1	1
1964,780137	49,41392694	49	0	1	1
1958,530137	55,68310502	55	0	2	1
1953,638128	60,68858447	60	1	0	0
1951,952283	62,56689498	62	0	1	1
1957,532877	57,00547945	57	0	1	1
1981,196804	33,47260274	33	0	0	0
1957,016438	57,80753425	57	0	2	1
1962,935845	51,92488584	51	0	2	1
1940	74,84155251	74	0	2	0
1956,310274	58,68584475	58	1	0	0
1969,474201	45,5586758	45	0	0	0
1939,083333	76,03013699	76	0	0	0
1953,416667	61,72968037	61	0	2	1
1954,565753	60,68310502	60	1	2	0
1939,412785	76,00388128	76	1	2	1
1953,243379	62,23356164	62	0	0	0
1952,713242	62,78287671	62	0	2	1
1950,315753	65,33333333	65	0	0	0
1971,882648	43,86073059	43	0	1	1
1955,005479	60,79657534	60	0	1	0

Postoperative Smoking (0=	Postoperative Alcohol (0=no	Previous CHT-RT (0=no	Site (1=oral ca	Subsite (1=tongue
1	0	0	1	2
0	0	0	1	1
1	1	0	1	1
0	0	0	1	1
1	1	0	1	1
0	0	0	1	1
0	1	0	1	1
0	0	0	1	1
0	1	0	1	1
0	0	0	1	1
0	0	1	1	1
0	0	0	1	1
0	0	0	1+2	1+4 (synchronous t
1	0	0	1	1+2+3
1	1	0	1	2
0	0	2	1	1
1	1	0	1	1
0	0	0	1	1
0	0	1	1	1+3
0	0	0	1	1
1	1	0	1	1
0	0	0	1	1
0	0	2	1	1+2
0	1	2	1	2
0	1	2	1	1
1	0	0	1	1+3
0	0	0	1	1
0	0	0	1	1
0	0	0	1	1+2
0	0	0	1	1
0	0	3	2	4
0	0	1	1	2
1	0	0	1	1+2
0	0	0	1	1+2
0	1	0	1	2
0	0	0	1	1
1	1	0	1	1
0	0	0	1	1
0	0	0	1	1
0	0	0	1	1
0	0	3	1	1
0	0	1	1	1
0	0	0	1	1
0	0	3	1	1+2
0	0	0	1	1
0	0	0	1	1+2
0	0	0	1	2
0	0	0	1	1
0	0	0	1	1
0	1	0	1	1+2
0	0	0	1	1
0	0	0	1	1
1	0	0	1	1+2

Type of Glossectomy (Ans	Surgery on Pharynx/Larynx	Neck Dissection (0=no; 1=SND; 2=FN	Free Flap (1=forear
IIIb	0	1	1
IIIb	0	1	1
IIIb	0	1+1	1
IIIb	0	2	1
IIIb	0	1	1
IIIb	0	3 (SAN)	1
IIIb	Oropharyngectomy + tonsillectomy	1	1
IIIb	0	1	1
IIIb	0	1+2	1
IIIb	0	2	1
IVa + segmental mandibule	0	1	4
IIIb	0	1	1
IIIb	Oropharyngectomy	2	1
IVa + segmental mandibule	0	1+1	4
IVb	0	1	3
IIIb	0	1	1
IIIb	0	1	1
IIIb	0	1	1
IIIb + marginal mandibuloto	0	0 (2004)	4
IIIb	0	2	1
IIIb	0	2	3
IIIb	0	2	1
IVa	0	2+2	1
IIIb	0	2+2	1
IIIb + marginal mandibuloto	0	1	1
IIIb + segmental mandibule	0	1+2	1
IIIb	0	1	1
IIIb	0	2	1
IVa	0	1+2	1
IIIb	0	1	1
V	0	1+1	3
IIIb	0	1	1
IIIb	0	2	1
IIIb	0	1+2	1
V	0	1+1	3
IIIb	Tonsillectomy	1	1
IIIb	0	1	1
IIIb	Tonsillectomy	2	1
IIIb	0	2	1
IIIb	0	1	1
IIIb	0	2	1
V	0	3 (IJV)	3
IIIb	0	2	1
IIIb	0	1	1
IIIb	0	2	1
IIIb	0	2	1
IIIb	0	1	1
IIIb	0	2	1
IIIb	0	1	1
IVa	0	2+2	1
IIIb	0	2	1
IIIb	0	1	1
IIIb	0	2	1

Surgeon	Day of Su	Month of S	Year of S	Surgery	pTNM AJCC 20	Staging AJCC 20	pTNM AJCC 2018
RP	17	11	2010	2010,877169	pT2N0Mx	II	pT2N0M0
RP	7	12	2010	2010,933105	pT2N2bMx	IVA	pT2N2bM0
RP	17	1	2011	2011,043836	pT2N0Mx	II	pT3N0M0
RP	31	1	2011	2011,082192	pT2N0Mx	II	pT3N0M0
RP	28	6	2011	2011,490639	pT2N0Mx	II	pT3N0M0
RP	30	8	2011	2011,662785	pT2N2bMx	IVA	pT3N2bM0
RP	17	10	2011	2011,793836	pT3N2bMx	IVA	pT2N2bM0
RP	25	10	2011	2011,815753	pT2N0Mx	II	pT2N0M0
RP	5	12	2011	2011,927626	pT4aN1Mx	IVA	pT4aN1M0
RP	15	12	2011	2011,955023	pT2N2bMx	IVA	pT2N2bM0
RP	21	12	2011	2011,971461	rypT1N0Mx	I	rypT2N0M0
RP	10	1	2012	2012,024658	pT2N0Mx	II	pT3N0M0
RP	31	1	2012	2012,082192	pT1N0; pT1N0	I; I	pT1N0M0
RP	7	2	2012	2012,099772	pT4aN0Mx	IVA	pT4aN0M0
RP	6	3	2012	2012,180365	rpT4aN0Mx	IVA	rpT4aN0M0
RP	12	4	2012	2012,280137	ypT1N0Mx	I	ypT2N0M0
RP	17	4	2012	2012,293836	pT2N0Mx	II	pT3N0M0
RP	10	5	2012	2012,357991	pT2N0Mx	II	pT2N0M0
RP	29	5	2012	2012,410046	rypT3N0M0	III	rypT3N0M0
RP	5	6	2012	2012,427626	pT1N0Mx	I	pT2N0M0
RP	10	7	2012	2012,524658	pT2N0Mx	II	pT3N0M0
RP	4	9	2012	2012,674886	pT1N0Mx	I	pT1N0M0
RP	25	9	2012	2012,73242	pT2N2cMx	IVA	pT2N2cM0
RP	23	10	2012	2012,810274	ypT1N0Mx	I	ypT2N0M0
RP	8	11	2012	2012,852511	ypT2N0M0	II	ypT2N0M0
RP	15	11	2012	2012,871689	pT4aN1M0	IVA	pT4aN1M0
RP	13	12	2012	2012,949543	pT1N0Mx	I	pT2N0M0
RP	17	1	2013	2013,043836	pT1N0Mx	I	pT1N0M0
RP	24	1	2013	2013,063014	pT2N0Mx	II	pT2N0M0
RP	13	6	2013	2013,449543	pT2N2bMx	IVA	pT3N2bM0
RP	5	11	2013	2013,844292	ypT4aN0Mx	IVA	ypT4aN0Mx
RP	12	11	2013	2013,86347	ypT2N0Mx	II	ypT2N0M0
RP	19	11	2013	2013,882648	pT2N1M0	III	pT2N1M0
RP	11	3	2014	2014,194064	pT1N0Mx	I	pT1N0M0
RP	18	3	2014	2014,213242	pT2N0M0	II	pT3N0M0
RP	29	4	2014	2014,326712	pT1N0M0	I	pT3N0M0
RP	8	7	2014	2014,519178	pT2N0Mx	II	pT2N0M0
RP	15	7	2014	2014,538356	pT2N2bM0	IVA	pT3N2bM0
RP	2	9	2014	2014,669406	pT1N0Mx	I	pT1N0M0
RP	28	10	2014	2014,823973	pT2N1Mx	III	pT3N1M0
RP	11	11	2014	2014,860731	rypT2N0M0	II	rypT3N0M0
RP	4	11	2014	2014,841553	rypT4aN2cM0	IVA	rypT4aN2cM0
RP	30	12	2014	2014,996119	pT1N0M0	I	pT2N0M0
RP	13	1	2015	2015,032877	rypT1N0M0	I	rypT1N0M0
RP	12	2	2015	2015,11347	pT2N1Mx	III	pT2N1M0
RP	24	2	2015	2015,146347	pT4aN2bM0	IVA	pT4aN2bM0
RP	31	3	2015	2015,248858	pT1N0M0	I	pT2N0M0
RP	1	6	2015	2015,416667	rpT1N0M0	I	rpT3N0M0
RP	23	6	2015	2015,476941	rpT2N0M0	II	rpT2N0M0
RP	30	6	2015	2015,496119	pT3N2cM1	IVC	pT4aN3bM1
RP	25	8	2015	2015,649087	pT2N2bMx	IVA	pT3N2bM0
RP	29	9	2015	2015,743379	pT1N0M0	I	pT3N0M0
RP	20	10	2015	2015,802055	pT2N1M0	III	pT3N1M0

Staging AJCC 20	Grading (1	Hospitalization (	Complications (	Complications	Treatment of Complication
II	1	17	0		
IVA	1	19	0		
III	1	30	1	Bleeding	Revision
III	1	14	0		
III	2	15	0		
IVA	2	13	0		
IVA	2	24	0		
II	2	15	0		
IVA	1	18	0		
IVA	1	26	0		
II	1	23	0		
III	1	14	1	Bleeding	Revision
I; I	3; 3	22	1	Near flap failure (blee	Microanastomosis revision
IVA	2	29	0		
IVA	1	15	0		
II	1	14	0		
III	3	16	0		
II	1	14	0		
III	2	17	1	Suture dehiscence	Local flap
II	3	14	0		
III	1	22	0		
I	1	17	0		
IVA	1	19	1	Bleeding	Revision
II	1	16	0		
II	1	18	0		
IVA	3	15	0		
II	1	16	1	Bleeding	Revision
I	1	20	1	Bleeding	Revision
II	1	24	0		
IVA	3	19	1	Bleeding	Revision
IVA	2	21	0		
II	1	14	0		
III	1	17	1	Bleeding	Revision
I	1	16	0		
III	2	22	0		
III	2	15	0		
II	2	18	0		
IVA	3	17	0		
I	1	16	1	Near flap failure (blee	Microanastomosis revision
III	2	17	1	Bleeding	Revision
III	2	16	0		
IVA	3	23	1	Salivary fistula	Conservative approach
II	1	14	0		
I	1	19	0		
III	1	16	0		
IVA	2	18	0		
II	2	17	0		
III	2	18	1	Bleeding	Revision
II	1	17	0		
IVC	2	15	0		
IVA	2	15	0		
III	2	17	0		
III	2	22	0		

Date of Treatment	Adjuvant Therapy (0=no; 1=l	CHT-RT	Follow-up (0=c
	2	CHT: TAXIT (Docetaxel, Cis	0
	0		2
19/1/11	0		0
	0		0
	0		1
	2	CHT: Al Sarraf (Cisplatin 75r	2
	0		0
	0		0
	3		2
	3	CHT: CDDP 50mg; RT: 63G	1
	0		2
10/1/12	0		0
2/2/12	0		2
	0		0
	2		2
	0		1
	0		1
	0		1
26/11/12; 15/12/12	2		2
	0		1
	0		0
	0		0
26/09/12	3	RT: 56,1 Gy on T (33 fractio	0
	0		1
	0		1
	0		0
15/12/2012	0		0
17/1/13	0		2
	0		2
17/06/2013	3	CHT (Platin); RT (60Gy on T	0
	0		2
	0		2
21/11/2013	0		1
	0		1
	0		1
	0		1
	0		1
	3	RT: 50,4Gy on N (28 fraction	1
02/09/14	0		1
02/11/14	0		2
	0		1
	0		2
	0		1
	0		1
	0		2
	3		1
	0		1
03/06/2015	0		1
	0		1
	0		2
	1		2
	1		1
	0		1

Day of Last Follow-u	onth of Last Follow-u	ear of Last Follow-u	Stat	Condition in Last Fo	Recurrenc
24	11	2015	2015,896347	**NED**	0
4	2	2011	2011,091553	**DOD**	1
4	8	2017	2017,591553	**NED**	0
1	2	2014	2014,083333	**NED**	0
10	11	2017	2017,857991	**NED**	0
10	1	2013	2013,024658	**DOD**	1
9	6	2016	2016,438584	**NED**	0
25	11	2016	2016,899087	**NED**	0
1	9	2014	2014,666667	**DOD**	1
16	12	2016	2016,957763	**NED**	0
6	3	2015	2015,180365	**NED**	0
27	4	2018	2018,321233	**NED**	0
17	1	2016	2016,043836	**DOC**	0
16	11	2017	2017,874429	**NED**	0
2	10	2012	2012,75274	**DOD**	1
15	6	2017	2017,455023	**NED**	0
26	1	2018	2018,068493	**AWD**	0
20	9	2016	2016,718721	**NED**	0
1	12	2015	2015,916667	**DOD**	1
25	2	2016	2016,149087	**NED**	0
5	3	2013	2013,177626	**NED**	0
11	1	2018	2018,027397	**NED**	0
28	7	2017	2017,573973	**NED**	0
19	12	2017	2017,965982	**NED**	0
18	2	2016	2016,129909	**NED**	0
8	7	2016	2016,519178	**NED**	0
27	10	2017	2017,821233	**NED**	0
30	10	2014	2014,829452	**DOD**	0
19	7	2013	2013,549315	**DOC**	0
25	1	2018	2018,065753	**NED**	0
6	5	2015	2015,347032	**DOD**	1
13	7	2015	2015,532877	**DOC**	1
15	12	2017	2017,955023	**NED**	0
20	10	2017	2017,802055	**NED**	0
15	7	2018	2018,538356	**NED**	1
20	4	2018	2018,302055	**NED**	0
10	11	2017	2017,857991	**NED**	0
3	12	2015	2015,922146	**NED**	0
14	11	2017	2017,86895	**NED**	0
6	5	2015	2015,347032	**DOD**	1
5	11	2017	2017,844292	**NED**	0
12	12	2014	2014,946804	**DOD**	0
21	12	2016	2016,971461	**NED**	0
28	11	2017	2017,907306	**NED**	0
15	7	2015	2015,538356	**DOD**	1
5	12	2017	2017,927626	**NED**	0
16	1	2018	2018,041096	**NED**	0
15	4	2017	2017,288356	**AWD**	0
5	12	2017	2017,927626	**NED**	0
20	11	2015	2015,885388	**DOD**	1+2
16	6	2016	2016,457763	**DOD**	2
13	4	2018	2018,282877	**NED**	0
15	12	2017	2017,955023	**NED**	0

Recurrence	Rtnm	Staging	Day of Recurrence
Left cervical lymph nodes	rpTxN2cMx	IVA	4
Right cervical lymph nodes (08/02/2012); right cervical	rypTxN3M0	IVB	8
Right cervical lymph nodes	rypN3	IVB	24
Local	rcT4a	IVA	2
Local	rycT4bN0M0	IVB	2
Right floor of mouth	rycT4aNxMx	IVA	16
Floor of mouth, lung metastases	rcT4aM1	IVC	5
			1
Right base of tongue, bilateral cervical lymph nodes	rcT4aN2cM1	IVC	17
Right floor of mouth, mediastinum metastases	rcT4aN2bM1	IVC	15
Right cervical lymph nodes lung metastases	rycTxN2cM1	IVC	24
Local recurrence, bone metastases (C1)	rcT4aM1	IVC	
Lung and bone metastases (sternum, D11, L1)	rycM1	IVC	15

Month of Recurr	Year of Recurren	Stat Recurrence
2	2011	2011,091553
2	2012	2012,102511
2	2014	2014,146347
10	2012	2012,75274
4	2014	2014,25274
5	2014	2014,374429
6	2015	2015,427626
5	2018	2018,333333
3	2015	2015,210502
4	2015	2015,288356
1		
7	2015	2015,563014
11	2015	2015,830594
4	2016	2016,288356

Salvage therapy (0=none; 1=surgery; 2=RT; 3=CHT)	Local Control (0=
	0
1: SND	1
	0
	0
	0
1: right SND (09/02/2012); 3: CHT: TAXIT (Docetaxel 75mg/mq, Cisplatin 75mg/mq) (05/06/2	1
	0
	0
1: RND	1
	0
	0
	0
	0
	0
0	1
	0
	0
	0
0	1
	0
	0
	0
	0
	0
	0
	0
	0
	0
	0
	0
3	1
0	1
	0
	0
	0
	0
	0
	0
	0
2+3	1
	0
	1
	0
	0
3	1
	0
	0
3	1
	0
0	1
0	1
	0
	0

Second primary tumor (0=no; 1=synchronous; 2=metachron	Survival (0=alive, NE	Day of Death	Month of De	Year of De
0	0			
0	1		4	2011
0	0			
0	0			
2: type III cordetomy for vocal cord SCC (12/10/2012)	0			
0	1	31	1	2013
0	0			
0	0			
0	1	10	2	2015
2: scalp cutaneous SCC (2016)	0			
0	2	20	8	2015
0	0			
1: oropharynx SCC (31/01/2012); CHT for lung carcinoma (2015	2	28	8	2016
0	0			
0	1	20	1	2014
0	0			
2: CHT for piriform sinus SCC (01/2018)	3			
2: type Va cordectomy for vocal cord SCC (30/10/2012)	0			
0	1			2015
0	0			
0	0			
0	0			
0	0			
1: epiglottectomy for epiglottis SCC (19/09/2012)	0			
2: surgery fo palate SCC (01/04/2014)	0			
0	0			
0	0			
2: lung carcinoma	2	1	1	2015
0	2			
0	0			
0	1	11	5	2015
0	2	21	7	2015
0	0			
0	0			
0	0			
2: maxillectomy for retromolar area SCC (26/02/2018)	0			
0	0			
0	0			
0	0			
0	1	25	9	2015
0	0			
0	1	10	4	2015
0	0			
0	0			
0	1		8	2016
0	0			
0	0			
0	3			
0	0			
0	1	25	11	2015
0	1	24	8	2016
0	0			
0	0			

Comorbidites (N)	Time_UC	Time_Rec	Recurrenc	Tracheostom	ICU (days)	DSS	OS
0	5,019178082	5,019178082	0	7	1	**0**	**0**
1	0,158447489	0,158447489	1	7	0	**1**	**1**
0	6,547716895	6,547716895	0	7	0	**0**	**0**
0	3,001141553	3,001141553	0	7	0	**0**	**0**
6	6,367351598	6,367351598	0	7	0	**0**	**0**
1	1,361872146	1,361872146	1	7	0	**1**	**1**
1	4,644748858	4,644748858	0	7	0	**0**	**0**
4	5,083333333	5,083333333	0	10	0	**0**	**0**
0	2,739041096	2,739041096	1	10	0	**1**	**1**
4	5,002739726	5,002739726	0	7	0	**0**	**0**
4	3,20890411	3,20890411	0	15	0	**0**	**1**
0	6,296575342	6,296575342	0	7	1	**0**	**0**
4	3,961643836	3,961643836	0	7	3	**0**	**1**
0	5,774657534	5,774657534	0	7	0	0	0
3	0,572374429	0,572374429	1	7	0	**1**	**1**
4	5,174885845	5,174885845	0	7	0	**0**	**0**
0	5,774657534	5,774657534	1	7	0	**0**	**0**
0	4,360730594	4,360730594	0	7	0	**0**	**0**
3	3,506621005	3,506621005	1	14	0	**1**	**1**
2	3,721461187	3,721461187	0	7	0	**0**	**0**
1	0,652968037	0,652968037	0	7	0	**0**	**0**
1	5,352511416	5,352511416	0	7	0	**0**	**0**
2	4,841552511	4,841552511	0	19	0	**0**	**0**
0	5,155707763	5,155707763	0	7	0	**0**	**0**
1	3,27739726	3,27739726	0	7	0	**0**	**0**
1	3,647488584	3,647488584	0	7	0	**0**	**0**
3	4,871689498	4,871689498	0	7	0	**0**	**0**
3	1,785616438	1,785616438	1	7	0	**0**	**1**
3	0,48630137	0,48630137	0	7	0	**0**	**1**
0	4,616210046	4,616210046	0	7	0	**0**	**0**
1	1,502739726	1,502739726	1	21	1	**1**	**1**
2	1,669406393	1,669406393	1	7	0	**1**	**1**
1	4,072374429	4,072374429	0	7	0	**0**	**0**
0	3,607990868	3,607990868	0	7	0	**0**	**0**
0	4,325114155	4,120091324	1	7	0	**0**	**0**
4	3,975342466	3,975342466	0	7	0	**0**	**0**
6	3,338812785	3,338812785	0	8	0	**0**	**0**
0	1,383789954	1,383789954	0	7	0	**0**	**0**
0	3,199543379	3,199543379	0	7	0	**0**	**0**
0	0,523059361	0,523059361	1	7	0	**1**	**1**
2	2,983561644	2,983561644	0	7	0	**0**	**0**
4	0,105251142	0,105251142	0	7	0	**1**	**1**
0	1,975342466	1,975342466	0	7	0	**0**	**0**
4	2,874429224	2,874429224	0	7	0	**0**	**0**
3	0,424885845	0,424885845	1	7	0	**1**	**1**
1	2,781278539	2,781278539	0	7	0	**0**	**0**
0	2,792237443	2,792237443	0	7	0	**0**	**0**
2	1,871689498	1,871689498	1	7	0	**0**	**0**
2	2,450684932	2,450684932	0	7	0	**0**	**0**
0	0,389269406	0,389269406	1	7	0	**1**	**1**
1	0,808675799	0,808675799	1	7	0	**1**	**1**
1	2,539497717	2,539497717	0	7	0	**0**	**0**
0	2,152968037	2,152968037	0	7	0	**0**	**0**

ORFS	pT 2010	pT 2018	LRFS	DMFS	Perineural invasi	Lymphovascular	Grading
0	2	2	0	0	0	1	1
1	2	2	1	0	0	0	1
0	2	3	0	0	0	0	1
0	2	3	0	0	0	0	1
0	2	3	0	0	0	0	2
1	2	3	1	0	0	0	2
0	3	3	0	0	0	0	2
0	2	2	0	0	0	0	2
1	4	4	1	0	0	1	1
0	2	2	0	0	0	1	1
0	1	2	0	0	0	0	1
0	2	3	0	0	0	0	1
0	1	1	0	0	0	0	3
0	4	4	0	0	0	0	2
1	4	4	1	0	0	0	1
0	1	2	0	0	0	0	1
0	2	3	0	0	0	0	3
0	2	2	0	0	0	0	1
1	3	3	1	0	0	0	2
0	1	2	0	0	0	0	3
0	2	3	0	0	0	0	1
0	1	1	0	0	0	0	1
0	2	2	0	0	0	0	1
0	1	2	0	0	0	0	1
0	2	2	0	0	0	0	1
0	4	4	0	0	1	0	3
0	1	2	0	0	0	0	1
0	1	1	0	0	0	0	1
0	2	2	0	0	0	0	1
0	2	3	0	0	0	1	3
1	4	4	1	0	1	1	2
1	2	2	1	0	0	0	1
0	2	2	0	0	0	0	1
0	1	1	0	0	0	0	1
1	2	3	1	0	0	0	2
0	1	3	0	0	0	0	2
0	2	2	0	0	0	0	2
0	2	3	0	0	1	1	3
0	1	1	0	0	0	0	1
1	2	3	1	1	0	1	2
0	2	3	0	0	1	1	2
1	4	4	1	0	1	1	3
0	1	2	0	0	1	0	1
0	1	1	0	0	0	0	1
1	2	2	1	1	0	1	1
0	4	4	0	0	1	1	2
0	1	2	0	0	1	0	2
1	1	3	1	1	0	1	2
0	2	2	0	0	0	1	1
1	3	4	1	1	1	1	2
1	2	3	0	1	1	1	2
0	1	3	0	0	1	0	2
0	2	3	0	0	0	0	2

PNI-LVI (0=no; 1=	pN (0=pN0; 1=pN+)	pN1	pN2	pN3	RT	CHT
1	0	0	0	0	0	1
0	1	0	1	0	0	0
0	0	0	0	0	0	0
0	0	0	0	0	0	0
0	0	0	0	0	0	0
0	1	0	1	0	0	1
0	1	0	1	0	0	0
0	0	0	0	0	0	0
1	1	1	0	0	1	1
1	1	0	1	0	1	1
0	0	0	0	0	0	0
0	0	0	0	0	0	0
0	0	0	0	0	0	0
0	0	0	0	0	0	0
0	0	0	0	0	0	1
0	0	0	0	0	0	0
0	0	0	0	0	0	0
0	0	0	0	0	0	0
0	0	0	0	0	0	1
0	0	0	0	0	0	0
0	0	0	0	0	0	0
0	0	0	0	0	0	0
0	1	0	1	0	1	1
0	0	0	0	0	0	0
0	0	0	0	0	0	0
1	1	1	0	0	0	0
0	0	0	0	0	0	0
0	0	0	0	0	0	0
0	0	0	0	0	0	0
1	1	0	1	0	1	1
2	0	0	0	0	0	0
0	0	0	0	0	0	0
0	1	1	0	0	0	0
0	0	0	0	0	0	0
0	0	0	0	0	0	0
0	0	0	0	0	0	0
0	0	0	0	0	0	0
2	1	0	1	0	1	1
0	0	0	0	0	0	0
1	1	1	0	0	0	0
2	0	0	0	0	0	0
2	1	0	1	0	0	0
1	0	0	0	0	0	0
0	0	0	0	0	0	0
1	1	1	0	0	0	0
2	1	0	1	0	1	1
1	0	0	0	0	0	0
1	0	0	0	0	0	0
1	0	0	0	0	0	0
2	1	0	0	1	0	0
2	1	0	1	0	1	0
1	0	0	0	0	1	0
0	1	1	0	0	0	0

CHT-RT (0=no; 1=	Previous RT (0=no; 1=yes)	Time_Met	Recurence	ENE
1	0	5,019178082		0
0	0	0,158447489	Lymph node	0
0	0	6,547716895		0
0	0	3,001141553		0
0	0	6,367351598		0
1	0	1,361872146	Lymph node	0
0	0	4,644748858		0
0	0	5,083333333		0
2	0	2,739041096	Lymph node	0
2	0	5,002739726		0
0	1	3,20890411		0
0	0	6,296575342		0
0	0	3,961643836		0
0	0	5,774657534		0
1	0	0,572374429	Local	0
0	0	5,174885845		0
0	0	5,774657534		0
0	0	4,360730594		0
1	1	3,506621005	Local	0
0	0	3,721461187		0
0	0	0,652968037		0
0	0	5,352511416		0
2	0	4,841552511		0
0	0	5,155707763		0
0	0	3,27739726		0
0	0	3,647488584		0
0	0	4,871689498		0
0	0	1,785616438		0
0	0	0,48630137		0
2	0	4,616210046		0
0	1	1,502739726	Local	0
0	1	1,669406393	Local	0
0	0	4,072374429		0
0	0	3,607990868		0
0	0	4,325114155	Lymph node	0
0	0	3,975342466		0
0	0	3,338812785		0
2	0	1,383789954		0
0	0	3,199543379		0
0	0	0,523059361	Lymph nodes + m	0
0	1	2,983561644		0
0	1	0,105251142		0
0	0	1,975342466		0
0	1	2,874429224		0
0	0	0,424885845	Lymph nodes + m	0
2	0	2,781278539		0
0	0	2,792237443		0
0	0	1,5	Lymph nodes + m	0
0	0	2,450684932		0
0	0	0,389269406	Lymph nodes + m	1
1	0	0,808675799	Metastases	0
1	0	2,539497717		0
0	0	2,152968037		0

Lympho Nodes Ratio (LNR)	Previous Surgery alone			
0				
0,0238				
0				
0				
0				
0,2045				
0,0379				
0				
0,0115				
0,037				
0				
0				
0				
0				
0				
0				
0				
0				
0				
0				
0				
0,0841				
0				
0				
0,0244				
0				
0	1			
0				
0,0351				
0				
0				
0,0149				
0				
0				
0				
0				
0,0816				
0				
0,0222				
0				
0,0435				
0				
0				
0,0156				
0,0303				
0				
0				
0	1			
0,097				
0,0769				
0				
0,0126				

**54**	**MM**	1229/2015	4	12	1966
**55**	**PG**	933/2017	16	5	1956
**56**	**PF**	1300/2015	28	2	1959
**57**	**CS**	972/2017	25	12	1956
**58**	**GP**	93/2016	13	4	1949
**59**	**CAA**	111/2016	6	6	1960
**60**	**PP**	133/2016	19	10	1956
**61**	**PG**	131/2016	31	10	1964
**62**	**MA**	358/2016	4	6	1947
**63**	**FA**	396/2016	1	2	1944
**64**	**CG**	809/2016	28	2	1967
**65**	**CM**	979/2016	9	11	1979
**66**	**ME**	19/2017	22	9	1941
**67**	**SF**	167/2017	2	1	1966
**68**	**CF**	188/2017	3	6	1948
**69**	**MSA**	252/2017	29	4	1965
**70**	**CP**	254/2017	20	9	1943
**71**	**BMPA**	313/2017	16	12	1960
**72**	**CGP**	437/2017	16	8	1949
**73**	**VM**	441/2017	22	2	1963
**74**	**ES**	440bis/2017	25	9	1971
**75**	**SML**	553/2017	9	1	1972
**76**	**SML**	604/2017	26	12	1935
**77**	**CG**	624/2017	22	6	1938
**78**	**MG**	788/2017	20	12	1969
**79**	**PA**	809/2017	2	9	1954
**80**	**SME**	895/2017	26	12	1960
**81**	**FS**	992/2018	28	6	1943

1966,924886	48,95228311	48	1	0	1
1956,374429	59,54771689	59	0	2	1
1959,157306	56,81689498	56	0	2	1
1956,98242	59,04771689	59	1	2	0
1949,282877	66,82237443	66	0	0	0
1960,430365	55,69406393	55	1	0	2
1956,799315	59,34429224	59	0	2	1
1964,832192	51,33447489	51	0	2	1
1947,424886	68,78561644	68	1	0	0
1944,083333	72,33219178	72	1	0	0
1967,157306	49,65844749	49	0	1	2
1979,855251	37,11347032	37	0	0	0
1941,724201	75,30045662	75	1	0	0
1966,00274	51,14086758	51	0	1	1
1948,422146	68,74726027	68	0	2	1
1965,326712	51,91392694	51	0	1	1
1943,718721	73,53949772	73	0	0	0
1960,957763	56,3783105	56	1	1	0
1949,624429	67,82511416	67	0	0	0
1963,140868	54,31415525	54	0	1	1
1971,73242	45,73630137	45	1	2	0
1972,021918	45,54931507	45	1	0	0
1935,98516	81,64200913	81	1	0	0
1938,474201	79,18584475	79	0	2	0
1969,968721	47,82511416	47	0	1	0
1954,669406	63,16666667	63	0	1	2
1960,98516	56,90296804	56	1	1	1
1943,490639	74,5	74	1	1	0

0	0	0	1	1
0	0	0	1	2
0	1	0	1	2
0	0	0	1	2
0	0	0	1	1
0	0	0	1	1
0	0	0	1	1
0	0	0	1	2
0	0	0	1	1
0	0	0	1	1
0	0	0	1	1+2
0	0	0	1	1
0	0	0	1	1
1	1	3	1	1
0	0	0	1	1
0	0	0	1	1+4
0	0	0	1	1
0	0	0	1	1
0	0	3	1	1
0	0	0	1	1
0	0	0	1	1
0	0	0	1	1
0	0	0	1	1
0	0	0	1	1+2
0	0	0	1	1+2
0	0	0	1	1+2
0	0	0	1	1+2
0	0	0	1	1

IIIb	0	2	1
IIIb	0	1+1	1
IIIb	0	1+1	1
IIIb	0	1	1
IIIb	0	2	1
IIIb	0	2	1
IIIb	0	1	1
IIIb	0	1+1	1
IVa	0	1+2	1
IIIb	0	1	1
IIIb	0	1+2	1
IIIb	0	1	1
IIIb	0	2	1
IVb	Tonsillectomy	2+2	2
IIIb	0	1	2
IIIb	0	2	1
IIIb	0	1	2
IIIb	0	1	1
IVb	0	2	2
IVb	0	2+2	2
IIIb	0	1	1
IIIb	0	1	1
V	0	1+2	2
IIIb	0	0 (2008)	2
IIIb	0	1+1	1
IIIb	Tonsillectomy	3 (SCM + SAN)	2
IVb	0	1+2	2
IVa	0	1+1	1

RP	17	11	2015	2015,877169	pT2N2bM0	IVA	pT3N2bM0
RP	3	12	2015	2015,922146	pT1N0M0	I	pT2N0M0
RP	22	12	2015	2015,974201	pT4aN0M0	IVA	pT4aN0M0
RP	12	1	2016	2016,030137	pT4aN0M0	IVA	pT4aN0M0
RP	9	2	2016	2016,105251	pT2N2bMx	IVA	pT2N2bM0
RP	16	2	2016	2016,124429	pT2N0Mx; pT1N	II; I	(m)pT2N0M0; pT1
RP	23	2	2016	2016,143607	pT2N1Mx	III	pT3N1M0
RP	1	3	2016	2016,166667	pT4aN0Mx	IVA	pT4aN0M0
RP	17	3	2016	2016,210502	pT4aN2cMx	IVA	pT4aN2cM0
RP	31	5	2016	2016,415525	pT2N0Mx	II	pT3N0M0
RP	25	10	2016	2016,815753	pT2N2bM0	IVA	pT2N2bM0
RP	20	12	2016	2016,968721	pT2N1Mx	III	pT3N1M0
RP	10	1	2017	2017,024658	pT2N2bMx	IVA	pT2N2bM0
RP	23	2	2017	2017,143607	ypT4aN2cM0	IVA	ypT4aN2cM0
RP	2	3	2017	2017,169406	pT2N0M0	II	pT2N0M0
RP	28	3	2017	2017,240639	pT2N1M0; pT1a	III - I	pT2N1M0
RP	4	4	2017	2017,258219	pT2N0M0	II	pT2N0M0
RP	2	5	2017	2017,336073	pT1N1M0	III	pT3N1M0
RP	13	6	2017	2017,449543	rypT2N0M0	II	rypT2N0M0
RP	15	6	2017	2017,455023	pT4aN2cM0	IVA	pT4aN3bM0 ENE
RP	20	6	2017	2017,468721	(m)pT2N0Mx	II	(m)pT3N0M0
RP	27	7	2017	2017,571233	pT2N1M0	III	pT3N1(sn)M0
RP	17	8	2017	2017,627169	pT2N1M0	III	pT3N2aM0
RP	29	8	2017	2017,660046	pT2cN0M0	II	pT2cN0M0
RP	17	10	2017	2017,793836	pT2N0Mx	II	pT2N0Mx
RP	2	11	2017	2017,836073	pT4aN2aM0	IVA	pT4aN2aM0
RP	21	11	2017	2017,888128	pT2N1Mx	III	pT3N1M0
RP	28	12	2017	2017,990639	pT1N1Mx	III	pT3N1Mx

IVA	2	24	1	Near flap failure (thror	Microanastomosis revision
II	3	16	0		
IVA	1	21	1	Salivary fistula	Conservative approach
IVA	2	16	0		
IVA	2	17	0		
II; I	2; 1	18	0		
III	2	15	0		
IVA	2	30	1	Bleeding	Revision
IVA	2	28	1	Bleeding	Revision
III	2	20	1	Bleeding	Revision
IVA	2	21	0		
III	2	18	0		
IVA	2	18	1	Bleeding	Revision
IVA	2	21	0		
II	2	16	0		
III	2; 2	17	0		
II	3	40	1	Suture dehiscence	Debridement and suture
III	2	21	0		
II	2	20	0		
IVB	3	30	0		
III	2	14	0		
III	2	22	1	Suture dehiscence	Debridement and suture
IVA	2	9	0		
II	2	23	0		
II	2	15	0		
IVA	3	29	0		
III	3	32	0		
III	2	22	0		

19/11/15	3		2
	1	54 Gy (30 fractions)	1
31/12/15	0		1
	0		1
	1	RT: 54Gy (30 fractions)	1
	0		1
	1	RT: 54 Gy (30 fractions)	1
03/03/16	1	RT: 60Gy on T + 51Gy on N	1
27/05/2016	0		2
06/06/2016	0		1
	0		1
	1		1
15/01/2017	1		1
	0		1
	0		1
	0		1
09/05/2017	0		1
	0		1
	0		1
	0		2
	1	RT: 60Gy on T, 54Gy on T +	1
05/08/2017	0		1
	0		2
	0		1
	0		1
	0		1
	1		1
	1		1

15	9	2016	2016,705023	**DOD**	2
22	1	2018	2018,057534	**NED**	1
12	12	2017	2017,946804	**NED**	0
22	1	2018	2018,057534	**NED**	0
2	3	2018	2018,169406	**NED**	0
1	12	2017	2017,916667	**NED**	0
11	1	2018	2018,027397	**NED**	1
2	3	2018	2018,169406	**NED**	0
22	9	2016	2016,724201	**DOD**	1+2
23	11	2017	2017,893607	**NED**	0
19	1	2017	2017	**NED**	0
21	12	2017	2017,971461	**NED**	0
18	1	2018	2018,046575	**NED**	0
1	5	2018	2018,333333	**DOD**	1
22	12	2017	2017,974201	**NED**	0
28	7	2017	2017,573973	**NED**	0
23	1	2018	2018,060274	**NED**	0
22	11	2017	2017,890868	**NED**	0
21	11	2017	2017,888128	**NED**	0
28	7	2017	2017,573973	**DOD**	2
1	6	2018	2018,416667	**NED**	0
5	1	2018	2018,010959	**NED**	0
19	8	2017	2017,632648	**NED**	0
15	5	2018	2018,371689	**NED**	0
15	6	2018	2018,455023	**NED**	0
15	1	2018	2018,038356	**NED**	0
22	1	2018	2018,057534	**NED**	0
15	6	2018	2018,455023	**NED**	0

Bilateral lung metastases	rycM1	IVC	4
Right cervical lymph nodes	rypTxN2cM0	IVA	31
Muscolar tissue metastases (right cervical)	rcT0N0pM1	IVC	2
Floor of mouth, bilateral lung metastases	rcT4aM1	IVC	15
Tongue, right cervical lymph nodes	rycT4aN2cM0	IVA	6
Cerebral metastases	rcM1	IVC	1
			7

7	2016	2016,508219
10	2017	2017,832192
11	2016	2016,836073
9	2016	2016,705023
7	2017	2017,513699
9	2017	2017,666667
3	2018	2018,183105

3	1
1: SND	0
	0
	0
	0
	0
1: MRND	0
	0
3	1
	0
	0
	0
	0
3	1
	0
	0
	0
	0
	0
0	1
	0
	0
	0
	0
	0
	0
	0
	0

0	1	21	9	2016
0	0			
0	0			
2: type II glossectomy and SLNB for contralateral tongue SCC (1	0			
1: CHT-RT for rectal adenocarcinome (06_10/2016)	0			
0	0			
0	0			
0	0			
0	1	30	12	2016
0	0			
0	0			
0	0			
0	0			
0	1	1	7	2018
0	0			
0	0			
0	0			
0	0			
0	0			
0	1	29	11	2017
0	0			
0	0			
0	2	18	8	2017
0	0			
0	0			
0	0			
0	0			
0	0			

3	0,827853881	0,827853881	1	10	0	**1**	**1**
0	2,135388128	2,135388128	1	7	0	**0**	**0**
5	1,97260274	1,97260274	0	7	0	**0**	**0**
4	2,02739726	2,02739726	0	6	0	**0**	**0**
3	2,064155251	2,064155251	0	8	0	**0**	**0**
1	1,792237443	1,792237443	0	7	0	**0**	**0**
1	1,883789954	1,883789954	0	7	0	**0**	**0**
1	2,002739726	2,002739726	0	7	0	**0**	**0**
4	0,51369863	0,51369863	1	7	0	**1**	**1**
0	1,478082192	1,478082192	0	7	0	**0**	**0**
1	0,184246575	0,184246575	0	7	0	**0**	**0**
0	1,002739726	1,002739726	0	9	0	**0**	**0**
0	1,021917808	1,021917808	0	7	0	**0**	**0**
4	1,189726027	1,189726027	1	8	0	**1**	**1**
1	0,804794521	0,804794521	0	9	0	**0**	**0**
0	0,333333333	0,333333333	0	7	0	**0**	**0**
0	0,802054795	0,802054795	0	15	0	**0**	**0**
0	0,554794521	0,554794521	0	7	0	**0**	**0**
2	0,438584475	0,438584475	0	7	0	**0**	**0**
1	0,118949772	0,118949772	1	11	0	**1**	**1**
2	0,947945205	0,714383562	0	6	0	**0**	**0**
0	0,439726027	0,439726027	0	7	0	**0**	**0**
2	0,005479452	0,005479452	0	2	0	**0**	**1**
4	0,711643836	0,711643836	0	7	0	**0**	**0**
0	0,661187215	0,661187215	0	7	0	**0**	**0**
0	0,202283105	0,202283105	0	20	0	**0**	**0**
0	0,169406393	0,169406393	0	7	0	**0**	**0**
0	0,464383562	0,464383562	0	17	0	**0**	**0**

1	2	3	0	1	1	1	2
1	1	2	1	0	1	0	3
0	4	4	0	0	0	0	1
0	4	4	0	0	0	0	2
0	2	2	0	0	1	1	2
0	2	2	0	0	0	0	2
1	2	3	0	1	1	1	2
0	4	4	0	0	0	0	2
1	4	4	1	1	0	0	2
0	2	3	0	0	0	0	2
0	2	2	0	0	0	0	2
0	2	3	0	0	1	0	2
0	2	2	0	0	1	1	2
1	4	4	1	0	1	1	2
0	2	2	0	0	1	0	2
0	2	2	0	0	0	0	2
0	2	2	0	0	0	0	3
0	1	3	0	0	0	0	2
0	2	2	0	0	0	1	2
1	4	4	0	1	1	1	3
1	2	3	1	0	1	0	2
0	2	3	0	0	0	1	2
0	2	3	0	0	1	1	2
0	2	2	0	0	0	1	2
0	2	2	0	0	0	0	2
0	4	4	0	0	1	1	3
0	2	3	0	0	0	0	3
0	1	3	0	0	1	0	2

2	1	0	1	0	1	1
1	0	0	0	0	1	0
0	0	0	0	0	0	0
0	0	0	0	0	0	0
2	1	0	1	0	1	0
0	0	0	0	0	0	0
2	1	1	0	0	1	0
0	0	0	0	0	1	0
0	1	0	1	0	0	0
0	0	0	0	0	0	0
0	1	0	1	0	0	0
1	1	1	0	0	1	0
2	1	0	1	0	1	0
2	1	0	1	0	0	0
1	0	0	0	0	0	0
0	1	1	0	0	0	0
0	0	0	0	0	0	0
0	1	1	0	0	0	0
1	0	0	0	0	0	0
2	1	0	0	1	0	0
1	0	0	0	0	1	0
1	1 (sn)	1	0	0	0	0
2	1	0	1	0	0	0
1	0	0	0	0	0	0
0	0	0	0	0	0	0
2	1	0	1	0	0	0
0	1	1	0	0	1	0
1	1	1	0	0	1	0

2	0	0,7	Metastases	0
1	0	2,135388128	Lymph node	0
0	0	1,97260274		0
0	0	2,02739726		0
1	0	2,064155251		0
0	0	1,792237443		0
1	0	1,5	Metastases	0
1	0	2,002739726		0
0	0	0,51369863	Local + metastase	0
0	0	1,478082192		0
0	0	0,184246575		0
1	0	1,002739726		0
1	0	1,021917808		0
0	1	1,189726027	Lymph node	0
0	0	0,804794521		0
0	0	0,333333333		0
0	0	0,802054795		0
0	0	0,554794521		0
0	1	0,438584475		0
0	0	0,118949772	Metastases	1
1	0	0,947945205	Lymph node	0
0	0	0,439726027		0
0	0	0,005479452		1
0	0	0,711643836		0
0	0	0,661187215		0
0	0	0,202283105		0
1	0	0,169406393		0
1	0	0,464383562		0

0,0361				
0				
0				
0				
0,0408				
0				
0,0278				
0				
0,0727				
0				
0,0149				
0,0159				
0,0526				
0,0297				
0				
0,027				
0				
0,0192				
0				
0,223				
0				
0,0689				
0,0344				
0				
0,0161				
0,012				
0,0185				

